# Novel Opioids: Systematic Web Crawling Within the e-Psychonauts’ Scenario

**DOI:** 10.3389/fnins.2020.00149

**Published:** 2020-03-18

**Authors:** Davide Arillotta, Fabrizio Schifano, Flavia Napoletano, Caroline Zangani, Liam Gilgar, Amira Guirguis, John Martin Corkery, Eugenio Aguglia, Alessandro Vento

**Affiliations:** ^1^Department of Clinical and Experimental Medicine, Psychiatry Unit, University of Catania, Catania, Italy; ^2^Psychopharmacology, Drug Misuse and Novel Psychoactive Substances Research Unit, School of Life and Medical Sciences, University of Hertfordshire, Hatfield, United Kingdom; ^3^East London Foundation Trust (ELFT), Homerton University Hospital, London, United Kingdom; ^4^Department of Health Sciences, University of Milan, Milan, Italy; ^5^Gabalfa Clinic, Cardiff and Vale NHS Health Board, Cardiff, United Kingdom; ^6^Swansea University Medical School, Institute of Life Sciences, Swansea University, Singleton Park, Swansea, United Kingdom; ^7^Addictions’ Observatory (ODDPSS), Rome, Italy; ^8^School of Psychology, G. Marconi, Telematic University, Rome, Italy; ^9^Department of Mental Health, Rome, Italy

**Keywords:** psychonauts, novel psychoactive substance, novel synthetic opioids, fentanyl analogs, web crawling, drug misuse, prescribed drug misuse

## Abstract

**Background:**

A wide range of novel psychoactive substances (NPSs) are regularly searched and discussed online by e-psychonauts. Among NPSs, the range of prescription/non-prescription opioids (fentanyl and non-fentanyl analogs) and herbal derivatives currently represents a challenge for governments and clinicians.

**Methods:**

Using a web crawler (i.e., NPS.Finder^®^), the present study aimed at assessing psychonaut fora/platforms to better understand the online situation regarding opioids.

**Results:**

The open-web crawling/navigating software identified some 426 opioids, including 234 fentanyl analogs. Of these, 176 substances (162 were very potent fentanyls, including two ohmefentanyl and seven carfentanyl analogs) were not listed in either international or European NPS databases.

**Conclusion:**

A web crawling approach helped in identifying a large number, indeed higher than that listed by European/international agencies, of unknown opioids likely to possess a significant misuse potential. Most of these novel/emerging substances are still relatively unknown. This is a reason of concern; each of these analogs potentially presents with different toxicodynamic profiles, and there is a lack of docking, preclinical, and clinical observations. Strengthening multidisciplinary collaboration between clinicians and bioinformatics may prove useful in better assessing public health risks associated with opioids.

## Introduction

Novel psychoactive substances (NPSs) are substances that are not controlled by the United Nations (UN) 1961 Single Convention on Narcotic Drugs or by Psychotropic Substances Conventions (United Nations Office on Drugs and Crime [UNODC], 2019a). By definition, “novel” does not necessarily imply that a drug has been recently developed (Hassan et al., 2017); it may also refer to substances that have lately become popular and/or more widely available, constituting a reason of current or potential public health concern (Schifano et al., 2015). NPSs are mainly of synthetic origin and comprise different drug classes (Schifano et al., 2019b). Among them, over the recent years, there has been an increase in the appearance of novel synthetic opioids (NSOs) on the recreational drug market (Zawilska, 2017). NSOs are frequently used with other illegal or prescribed drugs (Pichini et al., 2018; Pérez-Mañá et al., 2018). Owing to a range of reasons, the non-medical use of opioids such as fentanyl analogs and a range of remaining prescription/non-prescription substances is spreading worldwide (Prekupec et al., 2017; Lovrecic et al., 2019) and is affecting the entire life span, from youngsters to the elderly (Huhn et al., 2018; Kelley-Quon et al., 2019). Opioids are among the most powerful analgesic drugs, but they are burdened by unwanted adverse effects, in particular the abuse liability and the respiratory depression, with the last being the primary cause of death from overdose (Valentino and Volkow, 2018; Algera et al., 2019; Varga et al., 2020). Further, the evidence suggests that opioids’ consumption impacts the *in utero* neuronal development and induces in humans long-lasting transgenerational changes in subsequent generations owing to epigenetic alterations (Gilardi et al., 2018). For these reasons, worldwide intensive endeavors are directed from academics, clinicians, and industries toward expanding, intensifying, and coordinating fundamental, translational, and clinical research with respect to opioid abuse. To this respect, there is strong focus at present on the study and development of abuse-deterrent opioid formulations (Pergolizzi et al., 2018). Despite the efforts of law enforcement and other agencies (European Monitoring Centre for Drugs and Drug Addiction [EMCDDA], 2019; United Nations Office on Drugs and Crime [UNODC], 2019c), an unknown number of substances are continuously manufactured, (illicitly) offered for sale, and inappropriately consumed (Davey et al., 2012; Orsolini et al., 2017). Some concerns are related as well to the lack of regulation relating to so-called home-brew opiates (Galanie et al., 2015; Oye et al., 2015).

### Number of Opioids Identified

By April 2019, the EMCDDA European Database on New Drugs (European Database on New Drugs [EDND], 2019) contained 751 entries, with 51 of them being classified as opioids. By February 2019, the UNODC listed a total of 892 substances in their NPS database (Unodc Early Warning Advisory on New Psychoactive Substances [UNODC EWA NPS], 2019), with 61 being NSOs. It could, however, be argued that the NPS scenario is much larger than that outlined by those substances that have been seized and formally identified by the European Union (EU) and the UN databases. Because the online NPS scenario typically predicts the real-life NPS market availability (Corazza et al., 2013; Schifano et al., 2015), identifying what is being discussed online by web-based NPS enthusiasts (e.g., the “e-psychonauts”) may well be of interest (Orsolini et al., 2015; Corkery et al., 2017).

Although data about the clinical, pharmacological, and toxicological characteristics of a number of opioid drugs have already been made available (Suzuki and El-Haddad, 2017; Armenian et al., 2018; Baumann et al., 2018; Beardsley and Zhang, 2018; Tabarra et al., 2019), there is a clear lack of knowledge relating to the number of novel substances offered to online/real-life customers (Kacinko and Papsun, 2019), also owing to the difficulties in detecting them in both interdiction and clinical settings (Abdulrahim and Bowden-Jones, 2018).

### Aims

The aim of the current research was to (a) identify and categorize the number of opioids collected by the NPS.Finder^®^ web crawler from a range of psychonaut, NPS-related, online sources; and (b) compare the NPS.Finder^®^ opioid list with related findings from the UNODC and the EMCDDA.

## Materials and Methods

### Identification of Substances

To facilitate the process of early recognition of the increasing dissemination of new substances online and the variability of information sources, a crawling/navigating software (i.e., “NPS.Finder^®^”) was designed to automatically scan the open/surface web for new/novel/emerging NPSs (for a thorough description of web crawling and data cleaning activities, see Schifano et al., 2019b). This software was designed to map on a 24/7 basis the large variety of psychoactive substances mentioned/discussed within a range of popular online psychonauts websites/fora. NPS.Finder^®^ was designed *de novo* (e.g., it was not designed by the authors; neither was it adapted from another program) by Damicom, an IT enterprise based in Rome (Italy), to extract a range of information regarding NPSs, including chemical and street names; chemical formula; three-dimensional images; and anecdotally reported clinical/psychoactive effects. These data were then automatically stored in an online, restricted-access/password-controlled database located within firewall protected, highly secure, and consistently performing servers. First, a number of proper piloting searches were carried out (see also Schifano et al., 2019b), and any new website of interest was added to the list, whose final version is attached as Table 1. Although the language most typically used in these websites was English, further languages analyzed by NPS.Finder^®^ included the following: Dutch, French, Turkish, Swedish, Spanish, German, Russian, and Italian_._ Afterward, a range of specific web scraper/crawler activities, to extract all accessible posts/entries from November 26, 2017 to May 31, 2019, were carried out. With the help an *ad hoc* check control panel, all data were manually and carefully analyzed by four medically/psychiatrically trained professionals (e.g., FN, DA, CZ, and LG). In this way, a full assessment and editing of each NPS.Finder^®^ data entry were carried out, and the range of unique opioids here commented was identified. Finally, using chemical structure identification and published related data, researchers assigned each molecule to its NPS drug class (Schifano et al., 2019b).

**TABLE 1:** NPS.Finder^®^ websites’ list; search activities carried out on the surface web only.

N	Website name
1	*Avalonmagicplants.com*
2	*Azarius.net*
3	*Bluelight.org*
4	*Bluemorphotours.com*
5	*Cannabis.net*
6	*Chemeurope.com*
7	*Committedpsychonaut.tumblr.com*
8	*Consolidated Index of Controlled Substances*
9	*Daath.hu/psychonauts*
10	*Dancesafe.org*
11	*Deviantart.com/psychonaut-a*
12	*Druglibrary.org*
13	*Drugs.tripsit.me*
14	*Drugs-forum.com*
15	*Drugs-plaza.com*
16	*Dutch-headshop.eu*
17	*Ecstasydata.org*
18	*Elephantos.com*
19	*Energycontrol.org*
20	*http://entheogen-network.com/forums/*
21	*Erowid.org*
22	*Eusynth.org*
23	*https://everything2.com/title/Psychonaut*
24	*Fungifun.org*
25	*Hedweb.com*
26	*Hipforums.com/forum*
27	*Isomerdesign.com*
28	*Knehnav.home.xs4all.nl*
29	*Kratomshop.com*
30	*Legal-high-inhaltsstoffe.de*
31	*Mindstates.org*
32	*Mycotopia.net*
33	*Natmedtalk.com*
34	*Npsproject.eu*
35	*Peyote.com/peyolink.html*
36	*Psychedelic-library.org*
37	*Psychonaut.ca*
38	*Psychonaut.fr*
39	*Psychonautdocs.com*
40	*Psychonautwiki.org*
41	*Psyconauts.tripod.com*
42	*Reddit.com and drug-related subreddits (e.g., Reddit.com/r/Psychonaut/; Reddit.com/r/shroomers/)*
43	*Shayanashop.com*
44	*Sjamaan.com*
45	*Tripzine.com*
46	*Tryptamind.com*
47	*Urban75.net*
48	*Wikipedia List of designer drugs*
49	*Zamnesia.com*

### Classification of Opioid Drugs

The web crawler-identified substances’ denominations were first searched in Medline/PubMed (PubMed, 2019) and in Google^®^/Google^®^ Scholar (Google, 2019; Google Scholar, 2019). The used terms were “opiates/opioids,” “opioids,” “novel synthetic opioids,” “fentanyl analogs,” “opioid receptors,” “mu-opioid receptor,” “delta-opioid receptor,” “kappa-opioid receptor,” “receptor binding affinity,” and “herbal compounds.” An initial screening was performed to classify the substances according to their main description (e.g., fentanyl vs. non-fentanyl analogs). For this purpose, both the PuCchem (PubChem, 2019) and ChEMBL (Davies et al., 2015; Gaulton et al., 2017; EMBL-EBI, 2019) were searched; and whenever possible, the International Union of Pure and Applied Chemistry (IUPAC) name was also used. For fentanyl analogs, to make sure that the index molecule was indeed a fentanyl, the IUPAC name proposed by “isomer design” (PiHKAL, 2019) was here considered. For a handful of substances, when there were errors in published IUPAC names, the ChemDraw approach, able to generate chemical names from structures and/or vice versa, was used (ChemDraw, 2019).

A further screening was performed to compare the NPS.Finder^®^ results with those reported by both the EMCDDA and the UNODC. More precisely, the search was performed on the following UN databases: International Narcotics Control Board (INCB) “Yellow List” (International Narcotics Control Board [INCB], 2019a); the Unodc Early Warning Advisory on New Psychoactive Substances [UNODC EWA NPS] (2019); and the fentanyl-related substances INCB report (International Narcotics Control Board [INCB], 2019a). For European data, the European Database on New Drugs [EDND] (2019) was accessed. To understand if an index opioid was currently being used as a prescribed medication, further screenings were carried out in the United Kingdom and United States lists of controlled substances (GOV.UK, 2017; DEA, 2019), and in the Anatomical Therapeutic Chemical/defined daily dose (ATC/DDD) list (Chen et al., 2012; WHO Collaborating Centre [WHOCC], 2016; WHO Collaborating Centre [WHOCC], 2019). The ATC substances were listed according to the ATC classification by WHO and manually searched in the ATC/DDD Index (WHO Collaborating Centre [WHOCC], 2019). In the ATC/DDD Index, most opioids are grouped with the code N02A (WHO Collaborating Centre [WHOCC], 2016).

## Results

### Identification and Classification of Opioid Drugs

After about 18 months of operation, the number of substances identified by the web crawler activities was 5,922. By the time of writing, some 4,204 unique NPSs were included in the database, and 1,718/5,922 (29.01%) remaining substances were found to be false positives or duplicates. The most common NPS mentioned in psychonaut fora included the following: psychedelic phenethylamines (30.1%; CI 95%: 28.7–31.5%); synthetic cannabimimetics (29.8%; CI 95%: 28.4–31.2%); and opioids (10.1%; CI 95%: 9.2–11.0%).

The opioids (*n* = 426) were then divided into two groups: *fentanyl analogs* (*n* = 234; 54.9%; CI 95%: 50.1–59.5%; Supplementary Table S1) and *miscellaneous opioids* (*n* = 192; 45.0%; CI 95%: 40.0–49.8%; Supplementary Tables S2A–C). The miscellaneous opioids group included the following: (a) all the prescribing opioids classified in the ATC/DDD Index (*n* = 48; 11.2% of 426; CI 95%: 8.6–14.6%); (b) the non-fentanyl analog opioids (*n* = 136; 31.9%; CI 95%: 27.6–36.5%); and (c) the herbal derivatives (*n* = 8; 1.8%; CI 95%: 0.9–3.6%) (Supplementary Tables S2A–C).

### Comparison With Novel Psychoactive Substance-/Opioid-Focused European and United Nations Databases

Current NPS.Finder^®^ results were compared with those pertaining to opioids listed in the EMCDDA and UN databases (Supplementary Tables 1, 2A–C). Overall, the NPS.Finder^®^ detected a larger number of opioids than was in the remaining databases. The opioids identified by *NPS.Finder*^®^ *only* were 176 chemically different substances. In particular, out of the 234 *fentanyl analogs*, 162 (69.2% of 234) were listed only in the NPS.Finder^®^; 7 (2.99%; e.g., 4-fluoroisobutyrfentanyl, acetylfentanyl, acrylfentanyl, butyrylfentanyl, furanylfentanyl, ocfentanil, and tetrahydrofuranylfentanyl) were listed in all databases, and the remaining 65 (27.78%) substances were reported in one or more of the EU or UN databases (i.e., INCB Yellow List; INCB “Fentanyl-related substances with no known legitimate use list”; UNODC EWA NPS; and EDND). Out of the 136 substances in the non-fentanyl analog opioid list, 14 (10.29%) were listed only in NPS.Finder^®^, and not in any of the following: INCB Yellow list (updated March 2019), EDND (April 2019), and UNODC EWA NPS (July 2019). The remaining 122 substances were reported both in the NPS.Finder^®^ and in at least one of the following: INCB Yellow list (updated March 2019), EDND (April 2019), or UNODC EWA NPS (July 2019).

## Discussion

To the best of our knowledge, an unprecedented list of opioid drugs with a possible recreational/misuse potential was generated by these open web-only crawling software activities, which were focusing on a range of psychonaut forum entries. For all these fentanyl/non-fentanyl/miscellaneous drugs, only limited levels of preclinical/clinical data are typically available (Deluca et al., 2012; Armenian et al., 2018; Frisoni et al., 2018; Gerace et al., 2018a; Ventura et al., 2018; European Database on New Drugs [EDND], 2019; Tabarra et al., 2019; United Nations Office on Drugs and Crime [UNODC], 2019b). Apart from the vast range of previously undescribed fentanyl analogs, NPS.Finder^®^ identified a further number of “novel” and potent/very potent, chemically diverse miscellaneous substances. This may suggest that psychonauts are attracted to a variety of drugs, which range from research chemicals and their derivatives to more “traditional” substances, including failed pharmaceuticals or old patents that have been “rediscovered” and marketed for their potential use as “recreational” substances (Corkery et al., 2018). At present, we cannot establish with certainty whether the opioids here identified are all, or in part, currently circulating in the community and are available for consumption. Indeed, the current paper focused on the e-psychonauts’ discussions only; these web-based drug enthusiasts, however, have been suggested to somehow represent the drug scenarios’ “trend setters” (Schifano et al., 2015, 2019b). Hence, a focus on web-based drug discussion may well be of interest to better assess, and possibly predict (Corazza et al., 2013), the international drug misuse concerns. Ongoing studies from our group will hopefully better identify the following: a) which of the e-psychonauts’ substances, including opioids, will make an entry into the future markets; and b) which is the time gap, for an index drug, between the start of the e-psychonauts’ interest and their actual identification on the international drug scenarios.

Different from both the UNODC and the EMCDDA, which report in their NPS databases only those substances that have been both seized from the community and chemically analyzed, it was unclear from here if the mentioned opioids have already been synthesized or not. For each mentioned opioid, however, the unique IUPAC name was here identified and reported, and those further sources here are considered (e.g., PubChem, ChEMBL, ChemDraw, and Isomer Design) confirmed that the substance was properly chemically characterized. Hence, one could argue that the rogue producers’ synthesis of any of the opioids here commented is indeed a real and distinct possibility (Kata et al., 2018; Financial Crimes Enforcement Network [FinCHEN], 2019; Pardo et al., 2019; Whitehouse, 2019).

Present results, highlighting a strong interest by psychonauts toward opioid drugs, are consistent with previous studies, which have analyzed the opioids’ debate both in social media settings (Kalyanam et al., 2017; Kim et al., 2017; Pandrekar et al., 2018; Li et al., 2019) and on the darknet (Mackey et al., 2018; Tzanetakis, 2018; Cunliffe et al., 2019).

### Fentanyl Analogs

Most (e.g., 55%) opioids identified here were fentanyl analogs. Although present findings do not necessarily confirm in any possible way these substances’ levels of use, they can still reflect the attention given by psychonauts to these drugs and help in explaining aspects of the current “opioid epidemic” (Kakko et al., 2019; Zhao, 2019). A few fentanyl analogs have been mentioned in the literature (Suzuki and El-Haddad, 2017; Zawilska, 2017; Misailidi et al., 2018; Lipiński et al., 2019), especially in terms of the acute clinical toxicity issues relating to their intake (Abdulrahim and Bowden-Jones, 2018). Vulnerable subjects can access online a large number of these substances (Suzuki and El-Haddad, 2017), without even being aware of what they are taking exactly (Ciccarone et al., 2017; Bardwell et al., 2019; McLean et al., 2019; Stein et al., 2019). Indeed, levels of related clinical toxicological information are sometimes available only postmortem (Giorgetti et al., 2017; Concheiro et al., 2018; D’Errico, 2018; Kraemer et al., 2019).

Apart from a range of some well-known fentanyl-related substances, two ohmefentanyl and seven carfentanyl analogs were here identified. It is of interest that the analgesic activity of ohmefentanyl in mice is 6,300 times more potent than that of morphine (Xu et al., 1985) and that carfentanyl has been reported in association with a number of fatalities (Wilcoxon et al., 2018). Given the wide range of common and street names available for each molecule, even small modifications occurring in the fentanyl family structure can lead to misidentification of the index drug (Akhondi et al., 2015) and potentially provoke clinically unexpected effects. It is also possible that some substances here commented by psychonauts included fentanyl precursors or metabolites (Wilde et al., 2019). Hence, it is particularly relevant, for the vast range of fentanyl analogs, to use unique codes such as those of IUPAC (Gaulton et al., 2017; Hähnke et al., 2018; PiHKAL, 2019).

### Miscellaneous: Non-fentanyl Compounds, Prescribing Opioids, Herbals, and Derivatives

The 136 non-fentanyl analog opioids identified in the NPS.Finder^®^ database belong to a range of pharmacological classes, and some 10.2% were not reported by the EU and UN databases. Several of these substances have been used in the past for research purposes and/or were never marketed. The present findings are consistent with the current “top” and “rising” posts on social networks like Reddit (Reddit, 2019d). Some of these substances, for example, BDPC/bromadol have already been described as being some 500 times more potent than morphine (Reddit, 2019a; Sharma et al., 2019; TripSit Factsheets, 2019a), whereas others, for example, embutramide, have been associated with suicidal intent (Lajtai et al., 2016). A range of remaining substances identified here have received little/no attention in the literature and include acetoxyketobemidone (e.g., an alternative to ketobemidone, a prescription analgesic that is scheduled in different countries; United Nations Office on Drugs and Crime [UNODC], 1954; Drugs-forum, 2007; Bluelight, 2019a; Reddit, 2019c); 6-methylenedihydrodesoxymorphine/6-MDDM (e.g., a semi-synthetic derivative of hydromorphone about 80 times more potent than morphine and associated with euphoriant effects; Bluelight, 2019b; TripSit Factsheets, 2019b; Reddit, 2019e); isopropyl-U-47700 (identified for the first time in 2018 and belonging to N-substituted benzamides and acetamide opioid analgesics, colloquially known as “U-compounds” or “U-drugs;” Krotulski and Logan, 2018; Sharma et al., 2019; Yin, 2019); and piperidylthiambutene (developed in the 1950s and equipotent to morphine; Adamson and Green, 1950).

NPS.Finder^®^ identified here virtually all the prescription opioids listed in the ATC/DDD Index. The psychonauts’ interest in commenting about these substances may be a reason of concern, because prescription opioid misuse is a challenging issue worldwide, especially so in both North America and Europe (van Amsterdam and van den Brink, 2015; Helmerhorst et al., 2017).

NPS.Finder^®^ identified as well a range of well-known opioid herbal compounds, including *Papaver somniferum* and some crude opiate extracts (e.g., granulate, tincture, and poppy seed tea); *Mitragyna speciosa*/kratom (but not mitragynine and 7-hydroxymitragynine; Fluyau and Revadigar, 2017; Graziano et al., 2017; Coe et al., 2019; Corkery et al., 2019); and *Salvia divinorum*. Although herkinorin (Ventura et al., 2018) was not identified in the current database, salvinorin B ethoxymethyl ether/“Symmetry” (e.g., an unusually potent synthetic salvinorin compound, potently binding to kappa opioid receptors; Peet and Baker, 2011; Erowid, 2015; Reddit, 2019b), salvinorin B methoxymethyl (a potent semi-synthetic derivative of salvinorin A; Baker et al., 2009; Peet and Baker, 2011; Reddit, 2019f; Zjawiony et al., 2019), and salvinorin A received here the attention of psychonauts. This may be a cause for concern, because salvinorin products’ psychoactive effects include perceptual disturbances, psychosis, irritability, and anxiety (Ventura et al., 2018).

Finally, it is interesting to note that a range of both endogenous (e.g., amphibian opioid peptides such as dermorphins and deltorphins) and food-derived (e.g., derivatives from milk and soya such as soymorphines and β-casomorphins) opioid peptides, possessing low levels of potency (Teschemacher et al., 1997; Negri et al., 2000; Ohinata et al., 2007; Liu and Udenigwe, 2018), were not mentioned in psychonaut fora.

### Comparison With European Union and United Nations Novel Psychoactive Substance-Related Databases

A total of 426 opioids (e.g., 234 fentanyl analogs and 192 non-fentanyl analogs) were identified. The number of NPS.Finder^®^ opioids is indeed higher than that listed by international agencies like the UNODC and the EMCDDA. By May 2019, the INCB identified 115 opioids (International Narcotics Control Board [INCB], 2019a); up to 2019, the UNODC EWA NPS listed 61 different opioids; by June 2018, the INCB listed 93 fentanyl-related substances with no known legitimate use (International Narcotics Control Board [INCB], 2019b); and by April 2019, the EMCDDA reported 51 opioids out a total of 749 EDND substances (European Database on New Drugs [EDND], 2019). There might be different reasons behind these inconsistencies. First, both the UN and EU agencies collect in their databases only those substances that are detected/seized and properly analyzed and most importantly reported, respectively, worldwide or in the European region. Furthermore, the NPS.Finder^®^ carried out a range of open web crawling identification activities focusing on a large range of psychonaut-based, specialized, multilingual, sources with a specific focus on new/old psychoactive substances of likely recreational interest. From this point of view, one could also argue that discussing a molecule on the web is not, *per se*, an indication that the index molecule is being/will be ingested by interested individuals. This can explain why the opioids common to all lists (e.g., the fentanyl analogs 4-fluoroisobutyrfentanyl, acetylfentanyl, acrylfentanyl, butyrylfentanyl, furanylfentanyl, ocfentanil, tetrahydrofuranylfentanyl, and the non-fentanyl analogs AH-7921 and U-47700) are only a small proportion of the total.

### Pharmacological and Clinical Considerations

Fentanyl, fentanyl analogs, and the remaining opioids here commented present as partial/full agonists, and with different affinity levels, at the mu, delta, and kappa opioid receptors. This may well suggest the existence of a possible vast range of ill-health consequences associated with these substances (Stein, 2016). However, a clear understanding of the clinical toxicity of each compound is at present problematic. In fact, although the *in vitro* pKi values/binding affinities, potency, and efficacy levels for a number of opioids are already available (World Health Organization [WHO], 2016; Baumann et al., 2018; World Health Organization [WHO], 2018), these may not provide enough information about the relative *in vivo* potency (Baumann et al., 2018). In fact, there might be variable effects on G-protein-coupled receptors, which could potentially give rise to a great diversity of intracellular consequences following the administration of different analogs with apparently similar pharmacodynamics (Smith et al., 2018). Furthermore, as highlighted using a molecular docking model, some substances are too structurally similar for the scoring function to distinguish between different analogs (Ellis et al., 2018, 2019). For some opioids, larger dosages of naloxone may be required to reverse the opioid toxidrome than needed in case of a typical heroin overdose (Armenian et al., 2018; Lovrecic et al., 2019). The greatest levels of concerns remain related to fentanyl analogs, because of their harmful potential (Schifano et al., 2019a), the continuous high incidence of emerging analogs on the markets over the last years (Schueler, 2017), and the difficulties in identifying them with analytical chemistry techniques (Gerace et al., 2018b; Morrow et al., 2019). Conversely, those substances here identified possessing a full/partial kappa opioid receptor agonist activities (including salvinorin A and its derivatives; tifluadom; and pethidine) are likely to be particularly attractive for opioid and other research chemical consumers prone to a recreational experimentation associated with complex psychoactive effects (Coffeen and Pellicer, 2019).

### Limitations

NPS.Finder^®^ crawled only on the open web during the present phase of its development. Future studies by our group will be focused on expanding drug searches on less accessible areas of the web such as the deep web and the darknet (Orsolini et al., 2017). A qualitative/netnographic approach (Loi et al., 2017; Wang, 2019) will be needed as well to better assess the levels of online information relating to the possible psychonauts’ preference between analogs and their motivation for use, that is, recreational or self-medication purposes. As previous studies have highlighted their importance in NPS-based studies (Deluca et al., 2012), next NPS.Finder^®^-based projects will need to focus as well on further languages, for example, Chinese, Japanese, and Arabic. Some consideration needs to be given as well to some opioids not having been reported by NPS.Finder^®^. The present findings, however, relate only to the psychonauts’ interest, who are typically debating/discussing/mentioning only those substances that are considered as “trendy.” Furthermore, it is possible that owing to fentanyl/non-fentanyl nomenclature issues, there may be discrepancies between the denomination of substances resulting from international/EU interdiction data and those mentioned by psychonauts.

## Conclusion

The web crawler activities may well possess the potential to identify a wide range of novel/previously undescribed NPSs, including opioids. The literature base regarding these substances is limited in terms of acute and long-term effects, adverse effects, abuse potential, and manufacturing/distribution in both the virtual and real markets. It is difficult for health professionals to keep up to date with the growing number of opioids being made available. Clinicians are not always aware of the risks relating to novel psychoactives’ intake, and, at the same time, they are not typically able to identify a potential NPS user (Simonato et al., 2013). This may be a reason for concern, especially for emergency professionals confronting acute, and at times dramatic, clinical situations that are suspected of being drug related but in which the standard urine specimen turns out to be negative (Guirguis et al., 2017). Hence, clinicians should be informed about the range of opioids, their idiosyncratic drug–drug combinations, and their medical risks (Orsolini et al., 2015). The availability of new digital technologies, and the current systematic web crawling activities, provided here better levels of knowledge about the emerging number of opioid derivatives. This may help in designing and developing a range of efficient opioid on−site screening and detection techniques (Guirguis et al., 2017) and in drafting potent opioids’ specific treatment and management guidelines.

Raising awareness and education among users, the general public, and frontline staff and the development of harm reduction techniques are of paramount importance to tackle the flood of opioids. The epidemic of opioid overdose is a complex problem that can only be addressed by concerted and multidisciplinary efforts. Indeed, *in silico*, *in vitro*, and *in vivo* studies could provide important findings; furthermore, it is deemed here essential to monitor the real-life scenarios through drug checking in interdiction, drug outpatient clinics, and critical care settings. The provided lists can be useful in prospective and forward-looking terms. More studies should aim at providing better levels of misusing drugs’ clinical pharmacological-related knowledge, so that properly tailored prevention strategies can be drawn up and made available.

## Data Availability Statement

The datasets generated for this study are available on request to the corresponding author.

## Author Contributions

FS and AV have conceived the idea of the manuscript and have coordinated the whole project. FN, CZ, DA, and LG have actually carried out the process of both data collection and systematization. DA performed the literature searches and the analysis of data and drafted the manuscript. FS and JC supervised the writing of the manuscript and contributed to the final version of the manuscript. FS and EA approved the final content of the manuscript. JC provided data from the EMCDDA and UNODC databases for the purposes of this research. FS, JC, and AG have provided relevant epidemiological data and have contributed as well to the drafting of the manuscript itself.

## Conflict of Interest

The authors declare that the research was conducted in the absence of any commercial or financial relationships that could be construed as a potential conflict of interest.
