# Supplementary material for: Novel Opioids: Systematic Web Crawling Within the e-Psychonauts’ Scenario
Source: Front Neurosci. 2020 Mar 18;14:149. doi: 10.3389/fnins.2020.00149 (PMC7093327; doi:10.3389/fnins.2020.00149)
Supplement: Supplementary file 1 [file Table_1.docx]

| **N** | ***NPS FINDER name (fentanyl analogues)*** | ***other names*** | ***IUPAC (isomerdesign)*** | ***INCB Yellow list (March 2019)*** | ***INCB (June 2018)*** | **EDND (April 2019)** | ***UNODC EWA NPS (July 2019)*** | ***Unique to NPS.Finder database*** |
| --- | --- | --- | --- | --- | --- | --- | --- | --- |
| 1 | **(Iso)Butyryl-F-Fentanyl N-Benzyl Analogue** | N-(1-benzylpiperidin-4-yl)-N-(4-fluorophenyl)-butanamide | N-(1-benzylpiperidin-4-yl)-N-(4-fluorophenyl)-butanamide | N | N | Y | Y | N |
| 2 | **2-Fluorofentanyl** | ortho-fluorofentanyl | N-[2-Fluoro-1-(2-phenylethyl)piperidin-4-yl]-N-phenylpropanamide | N | Y | Y | Y | N |
| 3 | **2-Isopropylfuranylfentanyl** | o-Isopropyl-furanylfentanyl | N-[1-(2-Phenylethyl)piperidin-4-yl]-N-[2-(propan-2-yl)phenyl]furan-2-carboxamide | N | Y | N | Y | N |
| 4 | **2-Methylfentanyl** |  | N-[2-Methyl-1-(2-phenylethyl)piperidin-4-yl]-N-phenylpropanamide | N | Y | N | N | N |
| 5 | **3-Allylfentanyl** |  | N-Phenyl-N-[1-(2-phenylethyl)-3-(prop-2-en-1-yl)piperidin-4-yl]propanamide | N | Y | N | N | N |
| 6 | **3-Fluorofentanyl** | meta-fluorofentanyl; NFEPP | N-[3-Fluoro-1-(2-phenylethyl)piperidin-4-yl]-N-phenylpropanamide | N | Y | Y | Y | N |
| 7 | **3-Methyl-Butyrylfentanyl** | 3-MBF | N-[3-Methyl-1-(2-phenylethyl)piperidin-4-yl]-N-phenylbutanamide | N | Y | N | N | N |
| 8 | **3-Methylcrotonylfentanyl** |  | N-phenyl-N-[1-(2-phenylethyl)-4-piperidinyl]-3-methylbut-2-enamide | N | N | Y | Y | N |
| 9 | **3-Methylfentanyl (3-Mf)** | Mefentanyl; 3-MF | N-[3-Methyl-1-(2-phenylethyl)piperidin-4-yl]-N-phenylpropanamide | Y | Y | N | N | N |
| 10 | **3-Methylthiofentanyl** |  | N-{3-methyl-1-[2-(thiophen-2-yl)ethyl]piperidin-4-yl}-N-phenylpropanamide | Y | Y | N | N | N |
| 11 | **3-Phenylpropanoylfentanyl** | β'-Phenylfentanyl | N,3-Diphenyl-N-[1-(2-phenylethyl)piperidin-4-yl]propanamide | N | Y | Y | Y | N |
| 12 | **4-Chloroisobutyrfentanyl** | p-Chloro-isobutyrylfentanyl; 4-Cl-iBF; p-Cl-iBF | N-(4-Chlorophenyl)-2-methyl-N-[1-(2-phenylethyl)piperidin-4-yl]propanamide | N | Y | Y | Y | N |
| 13 | **4'-Fluoro-Butyrylfentanyl** | 4-Fluoro-butyrylfentanyl; p-Fluoro-butyrylfentanyl; 4-FBF; p-FBF | N-(4-Fluorophenyl)-N-[1-(2-phenylethyl)piperidin-4-yl]butanamide | N | Y | N | Y | N |
| 14 | **4-Fluoro-Cyclopropylbenzylfentanyl** |  | N-(1-Benzylpiperidin-4-yl)-N-(4-fluorophenyl)cyclopropanecarboxamide | N | N | Y | Y | N |
| 15 | **4-Fluorofentanyl** | Parafluorofentanyl | N-[4-Fluoro-1-(2-phenylethyl)piperidin-4-yl]-N-phenylpropanamide | Y | Y | N | N | N |
| 16 | **4-Fluoroisobutyrfentanyl** | para-fluoroisobutyryl fentanyl; 4-F-iBF; 4-FiBF; 4F-iBF; FIBF; p-FIBF; p-FiBF | N-(4-fluorophenyl)-N-(1-phenethylpiperidin-4-yl)isobutyramide | Y | Y | Y | Y | N |
| 17 | **4'-Hydroxybutyrylfentanyl** | p-Hydroxybutyrylfentanyl; 4-HO-BF | N-(4-Hydroxyphenyl)-N-[1-(2-phenylethyl)piperidin-4-yl]butanamide | N | N | Y | N | N |
| 18 | **4-MeO-Butyrfentanyl** | 4-methoxy Butyryl fentanyl; p-methoxy Butyryl fentanyl; p-MeO Butyryl fentanyl; 4-MeO-BF | N-(4-methoxyphenyl)-N-(1-phenethylpiperidin-4-yl)butyramide | N | N | Y | N | N |
| 19 | **4''-Methoxyfentanyl** |  | N-{1-[2-(4-Methoxyphenyl)ethyl]piperidin-4-yl}-N-phenylpropanamide | N | Y | N | N | N |
| 20 | **4-Methoxymethylfentanyl** | R-30490 | N-[4-(Methoxymethyl)-1-(2-phenylethyl)piperidin-4-yl]-N-phenylpropanamide | N | Y | N | N | N |
| 21 | **4-Phenylfentanyl** |  | N-Phenyl-N-[4-phenyl-1-(2-phenylethyl)piperidin-4-yl]propanamide | N | Y | N | N | N |
| 22 | **Acetylbenzylfentanyl** |  | N-(1-benzyl-4-piperidyl)-N-phenyl-acetamide | N | N | Y | N | N |
| 23 | **Acetyl-Carfentanil** |  | Methyl 4-[acetyl(phenyl)amino]-1-(2-phenylethyl)piperidine-4-carboxylate | N | Y | N | N | N |
| 24 | **Acetylfentanyl** | acetyl fentanyl | N-Phenyl-N-[1-(2-phenylethyl)piperidin-4-yl]acetamide | Y | Y | Y | Y | N |
| 25 | **Acrylfentanyl** | Acryloylfentanyl; Egyptenyl | N-Phenyl-N-[1-(2-phenylethyl)piperidin-4-yl]prop-2-enamide | Y | Y | Y | Y | N |
| 26 | **Alpha-Methylfentanyl** | Alphamethylfentanyl; α-Methylfentanyl; AMF | N-Phenyl-N-[1-(1-phenylpropan-2-yl)piperidin-4-yl]propanamide | Y | Y | N | N | N |
| 27 | **Alpha-Methylfentanyl Butanamide Analogue** | BF; B-F | 2-methyl-N-phenyl-N-[1-(1-phenylpropan-2-yl)piperidin-4-yl]propanamide | N | Y | Y | N | N |
| 28 | **Alpha-Methylthiofentanyl** | α-Methyl-thiofentanyl | N-Phenyl-N-{1-[1-(thiophen-2-yl)propan-2-yl]piperidin-4-yl}propanamide | Y | Y | N | N | N |
| 29 | **A-Methylacetylfentanyl** | α-Methyl-acetylfentanyl | N-Phenyl-N-[1-(1-phenylpropan-2-yl)piperidin-4-yl]acetamide | N | Y | N | N | N |
| 30 | **Benzodioxole-Fentanyl** | Benzodioxolefentanyl | N-Phenyl-N-[1-(2-phenylethyl)piperidin-4-yl]-2H-1,3-benzodioxole-5-carboxamide | N | Y | Y | Y | N |
| 31 | **Benzofuranyl-Fentanyl** |  | N-{1-[2-(1-Benzofuran-5-yl)ethyl]piperidin-4-yl}-N-phenylpropanamide | N | Y | N | N | N |
| 32 | **Benzoylbenzylfentanyl** |  | N-phenyl-N-[1-(2-phenylethyl)-4-piperidinyl]-3-methylbut-2-enamide | N | Y | Y | Y | N |
| 33 | **Benzoylfentanyl** | Phenylfentanyl | N-Phenyl-N-[1-(2-phenylethyl)piperidin-4-yl]benzamide | N | Y | Y | Y | N |
| 34 | **Benzylfentanyl** | N-Benzylfentanyl; R-4129 | N-(1-Benzylpiperidin-4-yl)-N-phenylpropanamide | N | Y | Y | Y | N |
| 35 | **Beta-Hydroxyfentanyl** | β-Hydroxyfentanyl; Fentanol | N-[1-(2-Hydroxy-2-phenylethyl)piperidin-4-yl]-N-phenylpropanamide | Y | Y | N | N | N |
| 36 | **B-Hydroxy-3-Methyl-Thienylfentanyl** | β-Hydroxy-3-methyl-thienylfentanyl; β-Hydroxy-3-methyl-thiofentanyl | N-{1-[2-Hydroxy-2-(thiophen-2-yl)ethyl]-3-methylpiperidin-4-yl}-N-phenylpropanamide | Y | N | N | N | N |
| 37 | **B-Hydroxy-Thiofentanyl** | β-Hydroxy-thiofentanyl | N-{1-[2-Hydroxy-2-(thiophen-2-yl)ethyl]piperidin-4-yl}-N-phenylpropanamide | N | Y | N | Y | N |
| 38 | **B-Methylfentanyl** | β-Methylfentanyl | N-Phenyl-N-[1-(2-phenylpropyl)piperidin-4-yl]propanamide | N | Y | N | N | N |
| 39 | **Brifentanil** | A-3331 | N-{1-[2-(4-Ethyl-5-oxo-4,5-dihydro-1H-tetrazol-1-yl)ethyl]-3-methylpiperidin-4-yl}-N-(2-fluorophenyl)-2-methoxyacetamide | N | Y | N | N | N |
| 40 | **Butyrylfentanyl** | Butyrfentanyl; Bu-F; BUF; BF | N-Phenyl-N-[1-(2-phenylethyl)piperidin-4-yl]butanamide | Y | Y | Y | Y | N |
| 41 | **Carfentanil** | carfentanyl; Wildnil | Methyl 1-(2-phenylethyl)-4-[phenyl(propanoyl)amino]piperidine-4-carboxylate | Y | N | Y | Y | N |
| 42 | **Crotonylfentanyl** |  | (2E)-N-Phenyl-N-[1-(2-phenylethyl)piperidin-4-yl]but-2-enamide | N | Y | N | Y | N |
| 43 | **Cyclohexylfentanyl** |  | N-Phenyl-N-[1-(2-phenylethyl)piperidin-4-yl]cyclohexanecarboxamide | N | Y | N | Y | N |
| 44 | **Cyclopentyl-Fentanyl** | Cyclopentylfentanyl | N-Phenyl-N-[1-(2-phenylethyl)piperidin-4-yl]cyclopentanecarboxamide | N | Y | Y | Y | N |
| 45 | **Cyclopropylfentanyl** |  | N-Phenyl-N-[1-(2-phenylethyl)piperidin-4-yl]cyclopropanecarboxamide | N | Y | Y | Y | N |
| 46 | **Despropionyl-2-Fluoro Fentanyl** | Despropionyl-2-Fluorofentanyl | N-(2-fluorophenyl)-1-phenethylpiperidin-4-amine | N | Y | Y | Y | N |
| 47 | **Furanylbenzylfentanyl** |  | N-(1-Benzylpiperidin-4-yl)-N-phenylfuran-2-carboxamide | N | N | Y | Y | N |
| 48 | **Furanylfentanyl** | 2-Furanylfentanyl; Fu-F; FUF | N-Phenyl-N-[1-(2-phenylethyl)piperidin-4-yl]furan-2-carboxamide | Y | Y | Y | Y | N |
| 49 | **Furanyl-Norfentanyl** |  | N-Phenyl-N-(piperidin-4-yl)furan-2-carboxamide | N | Y | N | N | N |
| 50 | **Isobutyrylfentanyl** | IBF | 2-Methyl-N-phenyl-N-[1-(2-phenylethyl)piperidin-4-yl]propanamide | N | Y | N | N | N |
| 51 | **Lofentanil** |  | Methyl 3-methyl-1-(2-phenylethyl)-4-[phenyl(propanoyl)amino]piperidine-4-carboxylate | N | Y | N | N | N |
| 52 | **Methoxyacetyl-Fentanyl** | Methoxyacetylfentanyl; Methoxyacetyl-F; MAF | 2-Methoxy-N-phenyl-N-[1-(2-phenylethyl)piperidin-4-yl]acetamide | N | Y | Y | Y | N |
| 53 | **M-Fluoro-Methoxyacetylfentanyl** | m-Fluoro-methoxyacetylfentanyl; 3′-Fluoro-methoxyacetylfentanyl | N-(3-Fluorophenyl)-2-methoxy-N-[1-(2-phenylethyl)piperidin-4-yl]acetamide | N | N | Y | N | N |
| 54 | **Mirfentanyl** |  | N-[1-(2-Phenylethyl)piperidin-4-yl]-N-(pyrazin-2-yl)furan-2-carboxamide | N | Y | N | N | N |
| 55 | **M-Methylfentanyl** | m-Methylfentanyl; 3′-Methylfentanyl | N-(3-Methylphenyl)-N-[1-(2-phenylethyl)piperidin-4-yl]propanamide | N | Y | N | N | N |
| 56 | **N-Methyl-Carfentanil** | R-32395 | Methyl 1-methyl-4-[phenyl(propanoyl)amino]piperidine-4-carboxylate | N | Y | N | N | N |
| 57 | **N-Methylfentanyl** | N-Methyl norfentanyl | N-(1-Methylpiperidin-4-yl)-N-phenylpropanamide | N | Y | N | N | N |
| 58 | **Ocfentanil** | A-3217 | N-[1-(2-Phenylethyl)piperidin-4-yl]-N-(quinolin-8-yl)propanamide | Y | Y | Y | Y | N |
| 59 | **Ohmefentanyl** | β-hydroxy-3-methylfentanyl; OMF; RTI-4614-4 | N-[1-(2-hydroxy-2-phenylethyl)-3-methylpiperidin-4-yl]-N-phenylpropanamide | Y | Y | N | N | N |
| 60 | **O-Isopropyl-Furanylfentanyl** | 2′-Isopropyl-furanylfentanyl | N-[1-(2-Phenylethyl)piperidin-4-yl]-N-[2-(propan-2-yl)phenyl]furan-2-carboxamide | N | Y | N | N | N |
| 61 | **O-Methoxy-Furanylfentanyl** | 2′-Methoxy-furanylfentanyl; o-MeO-Fu-F | N-(2-Methoxyphenyl)-N-[1-(2-phenylethyl)piperidin-4-yl]furan-2-carboxamide | N | Y | N | N | N |
| 62 | **O-Methyl-Acetylfentanyl** | 2′-Methyl-acetylfentanyl; o-iPr-Fu-F | N-(2-Methylphenyl)-N-[1-(2-phenylethyl)piperidin-4-yl]acetamide | N | Y | Y | N | N |
| 63 | **P-Methoxyfentanyl** | p-Methoxyfentanyl; 4′-Methoxyfentanyl | N-(4-Methoxyphenyl)-N-[1-(2-phenylethyl)piperidin-4-yl]propanamide | N | Y | N | N | N |
| 64 | **P-Methoxy-Furanylfentanyl** | p-Methoxy-furanylfentanyl; 4′-Methoxy-furanylfentanyl | N-(4-Methoxyphenyl)-N-[1-(2-phenylethyl)piperidin-4-yl]furan-2-carboxamide | N | Y | N | N | N |
| 65 | **Tetrahydrofuranylfentanyl** | Tetrahydrofuran fentanyl; THF-F; THF-F | N-Phenyl-N-[1-(2-phenylethyl)piperidin-4-yl]oxolane-2-carboxamide | Y | Y | Y | Y | N |
| 66 | **Thenylfentanyl** |  | N-Phenyl-N-{1-[(thiophen-2-yl)methyl]piperidin-4-yl}propanamide | N | Y | N | N | N |
| 67 | **Thiofentanyl** | Thienylfentanil | N-Phenyl-N-{1-[2-(thiophen-2-yl)ethyl]piperidin-4-yl}propanamide | Y | Y | N | N | N |
| 68 | **Thiophenefentanyl** | Thiophene fentanyl (hydrochloride); 2-Thiofuranyl fentanyl; 2-Thiophenoyl fentanyl | N-(1-phenethylpiperidin-4-yl)-N-phenylthiophene-2-carboxamide, monohydrochloride | N | Y | Y | Y | N |
| 69 | **TMCP-F** | Tetramethylcyclopropyl-fentanyl | 2,2,3,3-Tetramethyl-N-phenyl-N-[1-(2-phenylethyl)piperidin-4-yl]cyclopropane-1-carboxamide | N | Y | N | N | N |
| 70 | **Trefentanil** | A-3665 | N-{1-[2-(4-Ethyl-5-oxo-4,5-dihydro-1H-tetrazol-1-yl)ethyl]-4-phenylpiperidin-4-yl}-N-(2-fluorophenyl)propanamide | N | Y | N | N | N |
| 71 | **Valerylfentanyl** | VF | N-Phenyl-N-[1-(2-phenylethyl)piperidin-4-yl]pentanamide | N | Y | Y | Y | N |
| 72 | **Α-Methyl-Butyrylfentanyl** | α-Methyl-butyrylfentanyl; α-Me-BF | N-Phenyl-N-[1-(1-phenylpropan-2-yl)piperidin-4-yl]butanamide | N | Y | N | N | N |
| 73 | **2',2''-Difluorofentanyl** | 2′,2″-DFF; 2″-Fluoro-o-Fluorofentanyl | N-(2-Fluorophenyl)-N-{1-[2-(2-fluorophenyl)ethyl]piperidin-4-yl}propanamide | N | N | N | N | **Y** |
| 74 | **2,3-Secofentanyl** |  | N-{4-[Methyl(2-phenylethyl)amino]butan-2-yl}-N-phenylpropanamide | N | N | N | N | **Y** |
| 75 | **2'-Fluoro-Butyrylfentanyl** | o-Fluoro-butyrylfentanyl | N-(2-Fluorophenyl)-N-[1-(2-phenylethyl)piperidin-4-yl]butanamide | N | N | N | N | **Y** |
| 76 | **2'-Fluoro-Isobutyrylfentanyl** | o-Fluoro-isobutyrylfentanyl; 2-FIBF | N-(2-Fluorophenyl)-2-methyl-N-[1-(2-phenylethyl)piperidin-4-yl]propanamide | N | N | N | N | **Y** |
| 77 | **2-Methyl Carfentanil** |  | Methyl 4-[(2-methylphenyl)(propanoyl)amino]-1-(2-phenylethyl)piperidine-4-carboxylate | N | N | N | N | **Y** |
| 78 | **3,3-Dimethylfentanyl** |  | N-[3,3-Dimethyl-1-(2-phenylethyl)piperidin-4-yl]-N-phenylpropanamide | N | N | N | N | **Y** |
| 79 | **3,5-Dimethyl-Cyclopropylfentanyl** |  | N-[3,5-Dimethyl-1-(2-phenylethyl)piperidin-4-yl]-N-phenylcyclopentanecarboxamide | N | N | N | N | **Y** |
| 80 | **3,5-Dimethylfentanyl** |  | N-[3,5-Dimethyl-1-(2-phenylethyl)piperidin-4-yl]-N-phenylpropanamide | N | N | N | N | **Y** |
| 81 | **3′-4′-Methylenedioxyfentanyl** | MD-F | N-(2H-1,3-Benzodioxol-5-yl)-N-[1-(2-phenylethyl)piperidin-4-yl]propanamide | N | N | N | N | **Y** |
| 82 | **3′-Me-4F-iBF** |  | N-(4-Fluorophenyl)-2-methyl-N-{1-[2-(3-methylphenyl)ethyl]piperidin-4-yl}propanamide | N | N | N | N | **Y** |
| 83 | **3′-Methyl-Methoxyacetylfentanyl** | m-Methyl-methoxyacetylfentanyl | 2-Methoxy-N-(3-methylphenyl)-N-[1-(2-phenylethyl)piperidin-4-yl]acetamide | N | N | N | N | **Y** |
| 84 | **3'-4'-Dichloro-3''-Fluorofentanyl** |  | N-(3,4-Dichlorophenyl)-N-{1-[2-(3-fluorophenyl)ethyl]piperidin-4-yl}propanamide | N | N | N | N | **Y** |
| 85 | **3-Ethylfentanyl** |  | N-[3-Ethyl-1-(2-phenylethyl)piperidin-4-yl]-N-phenylpropanamide | N | N | N | N | **Y** |
| 86 | **3'-Fluoro-Butyrylfentanyl** | m-Fluoro-butyrylfentanyl | N-(3-Fluorophenyl)-N-[1-(2-phenylethyl)piperidin-4-yl]butanamide | N | N | N | N | **Y** |
| 87 | **3'-Fluoro-Isobutyrylfentanyl** | m-Fluoro-isobutyrylfentanyl | N-(3-Fluorophenyl)-2-methyl-N-[1-(2-phenylethyl)piperidin-4-yl]propanamide | N | N | N | N | **Y** |
| 88 | **3-Methoxyfentanyl** |  | N-[3-Methoxy-1-(2-phenylethyl)piperidin-4-yl]-N-phenylpropanamide | N | N | N | N | **Y** |
| 89 | **3-Methyl Phenoxy Acetylfentanil** |  | N-[3-Methyl-1-(2-phenoxyethyl)piperidin-4-yl]-N-phenylacetamide | N | N | N | N | **Y** |
| 90 | **3-Methyl-Furanylfentanyl** | 3MFUF; TMFUF | N-[3-Methyl-1-(2-phenylethyl)piperidin-4-yl]-N-phenylfuran-2-carboxamide | N | N | N | N | **Y** |
| 91 | **4-(m-Hydroxyphenyl)Fentanyl** |  | N-[4-(3-Hydroxyphenyl)-1-(2-phenylethyl)piperidin-4-yl]-N-phenylpropanamide | N | N | N | N | **Y** |
| 92 | **4′-Chloro-Butyrylfentanyl** | 4Cl-Butyryl fentanyl; p-Chloro-butyrylfentanyl; p-CBF | N-(4-Chlorophenyl)-N-[1-(2-phenylethyl)piperidin-4-yl]butanamide | N | N | N | N | **Y** |
| 93 | **4′-Methylfentanyl** | p-Methylfentanyl; 4′-MF | N-(4-Methylphenyl)-N-[1-(2-phenylethyl)piperidin-4-yl]propanamide | N | N | N | N | **Y** |
| 94 | **4′-Methyl-Methoxyacetylfentanyl** | p-Methyl-methoxyacetylfentanyl | 2-Methoxy-N-(4-methylphenyl)-N-[1-(2-phenylethyl)piperidin-4-yl]acetamide | N | N | N | N | **Y** |
| 95 | **4′-Methyl-Tetrahydrofuranylfentanyl** | p-Methyl-tetrahydrofuranylfentanyl | N-(4-Methylphenyl)-N-[1-(2-phenylethyl)piperidin-4-yl]oxolane-2-carboxamide | N | N | N | N | **Y** |
| 96 | **4″-Nitrofentanyl** |  | N-{1-[2-(4-Nitrophenyl)ethyl]piperidin-4-yl}-N-phenylpropanamide | N | N | N | N | **Y** |
| 97 | **4-Anilino-N-phenylethyl-4-piperidine** | 4-ANPP; 1-Phenethyl-N-phenylpiperidin-4-amine; | N-phenyl-1-(2-phenylethyl)piperidin-4-amine | N | N | N | N | **Y** |
| 98 | **4''-Bromo-Ohmefentanyl** |  | N-{1-[2-(4-Bromophenyl)-2-hydroxyethyl]-3-methylpiperidin-4-yl}-N-phenylpropanamide | N | N | N | N | **Y** |
| 99 | **4'-Chloro-Cyclobutylfentanyl** | p-Chloro-cyclobutylfentanyl | N-(4-Chlorophenyl)-N-[1-(2-phenylethyl)piperidin-4-yl]cyclobutanecarboxamide | N | N | N | N | **Y** |
| 100 | **4'-Chloro-Cyclopentylfentanyl** | p-Chloro-cyclopentylfentanyl | N-(4-Chlorophenyl)-N-[1-(2-phenylethyl)piperidin-4-yl]cyclopentanecarboxamide | N | N | N | N | **Y** |
| 101 | **4'-Chloro-Cyclopropylfentanyl** | p-Chloro-cyclopropylfentanyl; 4′-Cl-Cyclopropyl-F | N-(4-Chlorophenyl)-N-[1-(2-phenylethyl)piperidin-4-yl]cyclopropanecarboxamide | N | N | N | N | **Y** |
| 102 | **4'-Chlorofentanyl** | p-Chlorofentanyl; p-CF | N-(4-Chlorophenyl)-N-[1-(2-phenylethyl)piperidin-4-yl]propanamide | N | N | N | N | **Y** |
| 103 | **4'-Fluoro-Acetylfentanyl** | p-Fluoro-acetylfentanyl; p-F-AF | N-(4-Fluorophenyl)-N-[1-(2-phenylethyl)piperidin-4-yl]acetamide | N | N | N | N | **Y** |
| 104 | **4'-Fluoro-Acrylfentanyl** | p-Fluoro-acrylfentanyl | N-(4-Fluorophenyl)-N-[1-(2-phenylethyl)piperidin-4-yl]prop-2-enamide | N | N | N | N | **Y** |
| 105 | **4''-Fluorofentanyl** |  | N-{1-[2-(4-Fluorophenyl)ethyl]piperidin-4-yl}-N-phenylpropanamide | N | N | N | N | **Y** |
| 106 | **4''-Fluoro-Ohmefentanyl** |  | N-{1-[2-(4-Fluorophenyl)-2-hydroxyethyl]-3-methylpiperidin-4-yl}-N-phenylpropanamide | N | N | N | N | **Y** |
| 107 | **4'-Fluoro-β-Hydroxy-Thiobutyrylfentanyl** | p-Fluoro-β-hydroxy-thiobutyrylfentanyl; BHT-FBF | N-(4-Fluorophenyl)-N-{1-[2-hydroxy-2-(thiophen-2-yl)ethyl]piperidin-4-yl}butanamide | N | N | N | N | **Y** |
| 108 | **4-Methyl Furanyl Fentanyl** | p-methyl Furanyl fentanyl; para-methyl Fu-F; p-methyl Fu-F; 4-methyl Fu-F | N-(1-phenethylpiperidin-4-yl)-N-(p-tolyl)furan-2-carboxamide, monohydrochloride | N | N | N | N | **Y** |
| 109 | **4''-Methyl-Acetylfentanyl** | 4-methylphenethylacetylfentanyl | N-{1-[2-(4-Methylphenyl)ethyl]piperidin-4-yl}-N-phenylacetamide | N | N | N | N | **Y** |
| 110 | **4''-Methylfentanyl** |  | N-{1-[2-(4-Methylphenyl)ethyl]piperidin-4-yl}-N-phenylpropanamide | N | N | N | N | **Y** |
| 111 | **4-Methylfentanyl** |  | N-[4-Methyl-1-(2-phenylethyl)piperidin-4-yl]-N-phenylpropanamide | N | N | N | N | **Y** |
| 112 | **A,3-Dimethylfentanyl** | α,3-Dimethylfentanyl | N-[3-Methyl-1-(1-phenylpropan-2-yl)piperidin-4-yl]-N-phenylpropanamide | N | N | N | N | **Y** |
| 113 | **A'-Methoxyfentanyl** | α′-Methoxyfentanyl | 2-Methoxy-N-phenyl-N-[1-(2-phenylethyl)piperidin-4-yl]propanamide | N | N | N | N | **Y** |
| 114 | **A-Methyl-Acrylfentanyl** | α-Methyl-acrylfentanyl; Acryloyl-α-methylfentanyl | N-Phenyl-N-[1-(1-phenylpropan-2-yl)piperidin-4-yl]prop-2-enamide | N | N | N | N | **Y** |
| 115 | **A'-Methyl-Butyrylfentanyl** | α′-Methyl-butyrylfentanyl | 2-Methyl-N-phenyl-N-[1-(2-phenylethyl)piperidin-4-yl]butanamide | N | N | N | N | **Y** |
| 116 | **A-Methyl-P-Fluorofentanyl** | α-Methyl-p-fluorofentanyl; AM-p-F-F | N-(4-Fluorophenyl)-N-[1-(1-phenylpropan-2-yl)piperidin-4-yl]propanamide | N | N | N | N | **Y** |
| 117 | **B-Hydroxy-Carfentanil** | β-Hydroxy-carfentanil | Methyl 1-(2-hydroxy-2-phenylethyl)-4-[phenyl(propanoyl)amino]piperidine-4-carboxylate | N | N | N | N | **Y** |
| 118 | **B-Hydroxy-P-Fluorofentanyl** | β-Hydroxy-p-fluorofentanyl; BH-p-F-F | N-(4-Fluorophenyl)-N-[1-(2-hydroxy-2-phenylethyl)piperidin-4-yl]propanamide | N | N | N | N | **Y** |
| 119 | **B-Hydroxy-Sufentanil** | β-Hydroxy-sufentanil | N-{1-[2-Hydroxy-2-(thiophen-2-yl)ethyl]-4-(methoxymethyl)piperidin-4-yl}-N-phenylpropanamide | N | N | N | N | **Y** |
| 120 | **Butyryl-Carfentanyl** |  | Methyl 4-[butanoyl(phenyl)amino]-1-(2-phenylethyl)piperidine-4-carboxylate | N | N | N | N | **Y** |
| 121 | **Butyrylremifentanil** |  | Methyl 4-[butanoyl(phenyl)amino]-1-(3-methoxy-3-oxopropyl)piperidine-4-carboxylate | N | N | N | N | **Y** |
| 122 | **Cyclobutylfentanyl** |  | N-Phenyl-N-[1-(2-phenylethyl)piperidin-4-yl]cyclobutanecarboxamide | N | N | N | N | **Y** |
| 123 | **Cyclopentenylfentanyl** |  | N-Phenyl-N-[1-(2-phenylethyl)piperidin-4-yl]cyclopent-1-ene-1-carboxamide | N | N | N | N | **Y** |
| 124 | **Ethyl [1-(2-Hydroxy-2-Phenylethyl)-3-Methylpiperidin-4-Yl]Phenylcarbamate** |  | Ethyl [1- (2-Hydroxy-2-Phenylethyl)-3-Methylpiperidin-4-Yl]Phenylcarbamate | N | N | N | N | **Y** |
| 125 | **Ethyl 1-(2-Hydroxy-2-Phenylethyl)-3-Methyl-4-[Phenyl (Propanoyl)Amino]Piperidine-4-Carboxylate** |  | Ethyl 1-(2-hydroxy-2-phenylethyl)-3-methyl-4-[phenyl(propanoyl)amino]piperidine-4-carboxylate | N | N | N | N | **Y** |
| 126 | **Fentranyl** | trans-Phenylcyclopropyl-norfentanyl | N-Phenyl-N-[1-(2-phenylcyclopropyl)piperidin-4-yl]propanamide | N | N | N | N | **Y** |
| 127 | **Fluoropentyl-Norcarfentanil** |  | Methyl 1-(5-fluoropentyl)-4-[phenyl(propanoyl)amino]piperidine-4-carboxylate | N | N | N | N | **Y** |
| 128 | **Isocarfentanyl** | 3-Carbomethoxyfentanyl; 3-Methoxycarbonylfentanyl | Methyl 1-(2-phenylethyl)-4-[phenyl(propanoyl)amino]piperidine-3-carboxylate | N | N | N | N | **Y** |
| 129 | **Isofentanyl** | N-Benzyl-3-methylfentanyl; 3-Methyl-benzylfentanyl | N-(1-Benzyl-3-methylpiperidin-4-yl)-N-phenylpropanamide | N | N | N | N | **Y** |
| 130 | **Isovaleroylfentanyl** |  | 3-Methyl-N-phenyl-N-[1-(2-phenylethyl)piperidin-4-yl]butanamide | N | N | N | N | **Y** |
| 131 | **Methacroylfentanyl** |  | 2-Methyl-N-phenyl-N-[1-(2-phenylethyl)piperidin-4-yl]prop-2-enamide | N | N | N | N | **Y** |
| 132 | **Methyl 1-(2-Hydroxy-2-Phenylethyl)-3-Methyl-4-[Phenyl (Propanoyl)Amino]Piperidine-4-Carboxylate** |  | Methyl 1-(2-hydroxy-2-phenylethyl)-3-methyl-4-[phenyl(propanoyl)amino]piperidine-4-carboxylate | N | N | N | N | **Y** |
| 133 | **Methyl 1-[ (2,3-Dihydro-1,4-Benzodioxin-2-Yl)Methyl]-4-[Phenyl (Propanoyl)Amino]Piperidine-4-Carboxylate** |  | Methyl 1-[(2,3-dihydro-1,4-benzodioxin-2-yl)methyl]-4-[phenyl(propanoyl)amino]piperidine-4-carboxylate | N | N | N | N | **Y** |
| 134 | **Methyl 1-[2-(2-Oxo-1,3-Benzoxazol-3 (2h)-Yl)Ethyl]-4-[Phenyl (Propanoyl)Amino]Piperidine-4-Carboxylate** |  | Methyl 1-[2-(2-oxo-1,3-benzoxazol-3(2H)-yl)ethyl]-4-[phenyl(propanoyl)amino]piperidine-4-carboxylate | N | N | N | N | **Y** |
| 135 | **Methyl 1-[2-(2-Oxo-2,3-Dihydro-1h-Indol-1-Yl)Ethyl]-4-[Phenyl (Propanoyl)Amino]Piperidine-4-Carboxylate** |  | Methyl 1-[2-(2-oxo-2,3-dihydro-1H-indol-1-yl)ethyl]-4-[phenyl(propanoyl)amino]piperidine-4-carboxylate | N | N | N | N | **Y** |
| 136 | **Methyl 1-[2-(3-Oxo-2,3-Dihydro-4h-1,4-Benzothiazin-4-Yl)Ethyl]-4-[Phenyl (Propanoyl)Amino]Piperidine-4-Carboxylate** |  | Methyl 1-[2-(3-oxo-2,3-dihydro-4H-1,4-benzothiazin-4-yl)ethyl]-4-[phenyl(propanoyl)amino]piperidine-4-carboxylate | N | N | N | N | **Y** |
| 137 | **Methyl 1-[2-(4-Methyl-1,3-Thiazol-5-Yl)Ethyl]-4-[Phenyl (Propanoyl)Amino]Piperidine-4-Carboxylate** |  | Methyl 1-[2-(4-methyl-1,3-thiazol-5-yl)ethyl]-4-[phenyl(propanoyl)amino]piperidine-4-carboxylate | N | N | N | N | **Y** |
| 138 | **Methyl 1-[2-Hydroxy-2-(Thiophen-2-Yl)Ethyl]-4-[Phenyl (Propanoyl)Amino]Piperidine-4-Carboxylate** |  | Methyl 1-[2-hydroxy-2-(thiophen-2-yl)ethyl]-4-[phenyl(propanoyl)amino]piperidine-4-carboxylate | N | N | N | N | **Y** |
| 139 | **Methyl 1-[2-Oxo-2-(Thiophen-2-Yl)Ethyl]-4-[Phenyl (Propanoyl)Amino]Piperidine-4-Carboxylate** |  | Methyl 1-[2-Oxo-2-(Thiophen-2-Yl)Ethyl]-4-[Phenyl (Propanoyl)Amino]Piperidine-4-Carboxylate | N | N | N | N | **Y** |
| 140 | **Methyl 1-{2-[(1-Methyl-1h-Imidazol-2-Yl)Sulfanyl]Ethyl}-4-[Phenyl (Propanoyl)Amino]Piperidine-4-Carboxylate** |  | Methyl 1-{2-[(1-Methyl-1h-Imidazol-2-Yl)Sulfanyl]Ethyl}-4-[Phenyl (Propanoyl)Amino]Piperidine-4-Carboxylate | N | N | N | N | **Y** |
| 141 | **Methyl 1-{2-[5-Methyl-2-(Methylsulfanyl)-6-Oxopyrimidin-1 (6h)-Yl]Ethyl}-4-[Phenyl (Propanoyl)Amino]Piperidine-4-Carboxylate** |  | Methyl 1-{2-[5-Methyl-2-(Methylsulfanyl)-6-Oxopyrimidin-1 (6h)-Yl]Ethyl}-4-[Phenyl (Propanoyl)Amino]Piperidine-4-Carboxylate | N | N | N | N | **Y** |
| 142 | **Methyl 4-[Phenyl (Propanoyl)Amino]-1-[2-(1h-Pyrazol-1-Yl)Ethyl]Piperidine-4-Carboxylate** |  | Methyl 4-[Phenyl (Propanoyl)Amino]-1-[2-(1h-Pyrazol-1-Yl)Ethyl]Piperidine-4-Carboxylate | N | N | N | N | **Y** |
| 143 | **Methyl 4-[Phenyl (Propanoyl)Amino]-1-[2-(1h-Pyrrol-1-Yl)Ethyl]Piperidine-4-Carboxylate** |  | Methyl 4-[Phenyl (Propanoyl)Amino]-1-[2-(1h-Pyrrol-1-Yl)Ethyl]Piperidine-4-Carboxylate | N | N | N | N | **Y** |
| 144 | **Methyl 4-[Phenyl (Propanoyl)Amino]-1-[2-(2h-Tetrazol-2-Yl)Ethyl]Piperidine-4-Carboxylate** |  | Methyl 4-[Phenyl (Propanoyl)Amino]-1-[2-(2h-Tetrazol-2-Yl)Ethyl]Piperidine-4-Carboxylate | N | N | N | N | **Y** |
| 145 | **Methyl 4-[Phenyl (Propanoyl)Amino]-1-[2-(Pyridin-2-Yl)Ethyl]Piperidine-4-Carboxylate** |  | Methyl 4-[Phenyl (Propanoyl)Amino]-1-[2-(Pyridin-2-Yl)Ethyl]Piperidine-4-Carboxylate | N | N | N | N | **Y** |
| 146 | **Methyl 4-[Phenyl (Propanoyl)Amino]-1-[2-(Thiophen-3-Yl)Ethyl]Piperidine-4-Carboxylate** |  | Methyl 4-[Phenyl (Propanoyl)Amino]-1-[2-(Thiophen-3-Yl)Ethyl]Piperidine-4-Carboxylate | N | N | N | N | **Y** |
| 147 | **N-(2-Fluorophenyl)-N-[1-(2-Hydroxy-2-Phenylethyl)-3-Methylpiperidin-4-Yl]Propanamide** |  | N-(2-Fluorophenyl)-N-[1-(2-hydroxy-2-phenylethyl)-3-methylpiperidin-4-yl]propanamide | N | N | N | N | **Y** |
| 148 | **N-(2-Fluorophenyl)-N-[1-(2-Phenylethyl)-4-(1,3-Thiazol-2-Yl)Piperidin-4-Yl]Propanamide** |  | N-(2-Fluorophenyl)-N-[1-(2-phenylethyl)-4-(1,3-thiazol-2-yl)piperidin-4-yl]propanamide | N | N | N | N | **Y** |
| 149 | **N-(2-Fluorophenyl)-N-[1-(2-Phenylethyl)-4-(Pyridin-2-Yl)Piperidin-4-Yl]Propanamide** |  | N-(2-Fluorophenyl)-N-[1-(2-phenylethyl)-4-(pyridin-2-yl)piperidin-4-yl]propanamide | N | N | N | N | **Y** |
| 150 | **N-(2-Fluorophenyl)-N-[4-Phenyl-1-(2-Phenylethyl)Piperidin-4-Yl]Propanamide** |  | N-(2-Fluorophenyl)-N-[4-phenyl-1-(2-phenylethyl)piperidin-4-yl]propanamide | N | N | N | N | **Y** |
| 151 | **N-(2-Fluorophenyl)-N-{1-[2-(1h-Pyrazol-1-Yl)Ethyl]-4-(Pyridin-2-Yl)Piperidin-4-Yl}Propanamide** |  | N-(2-Fluorophenyl)-N-{1-[2-(1H-pyrazol-1-yl)ethyl]-4-(pyridin-2-yl)piperidin-4-yl}propanamide | N | N | N | N | **Y** |
| 152 | **N-(2-Fluorophenyl)-N-{1-[2-(4-Methyl-1,3-Thiazol-5-Yl)Ethyl]-4-Phenylpiperidin-4-Yl}Propanamide** |  | N-(2-Fluorophenyl)-N-{1-[2-(4-Methyl-1,3-Thiazol-5-Yl)Ethyl]-4-Phenylpiperidin-4-Yl}Propanamide | N | N | N | N | **Y** |
| 153 | **N-(2-Fluorophenyl)-N-{4-(4-Methyl-1,3-Thiazol-2-Yl)-1-[2-(1h-Pyrazol-1-Yl)Ethyl]Piperidin-4-Yl}Propanamide** |  | N-(2-Fluorophenyl)-N-{4-(4-Methyl-1,3-Thiazol-2-Yl)-1-[2- (1h-Pyrazol-1-Yl)Ethyl]Piperidin-4-Yl}Propanamide | N | N | N | N | **Y** |
| 154 | **N-(2-Fluorophenyl)-N-{4-(4-Methyl-1,3-Thiazol-2-Yl)-1-[2-(4-Methyl-1,3-Thiazol-5-Yl)Ethyl]Piperidin-4-Yl}Propanamide** |  | N-(2-Fluorophenyl)-N-{4-(4-Methyl-1,3-Thiazol-2-Yl)-1-[2- (4-Methyl-1,3-Thiazol-5-Yl)Ethyl]Piperidin-4-Yl}Propanamide | N | N | N | N | **Y** |
| 155 | **N-(2-Fluorophenyl)-N-{4-(4-Methyl-1,3-Thiazol-2-Yl)-1-[2-(Thiophen-3-Yl)Ethyl]Piperidin-4-Yl}Propanamide** |  | N-(2-Fluorophenyl)-N-{4-(4-Methyl-1,3-Thiazol-2-Yl)-1-[2- (Thiophen-3-Yl)Ethyl]Piperidin-4-Yl}Propanamide | N | N | N | N | **Y** |
| 156 | **N-(2-Fluorophenyl)-N-{4-Phenyl-1-[2-(1h-Pyrazol-1-Yl)Ethyl]Piperidin-4-Yl}Propanamide** |  | N-(2-Fluorophenyl)-N-{4-Phenyl-1-[2-(1h-Pyrazol-1-Yl)Ethyl]Piperidin-4-Yl}Propanamide | N | N | N | N | **Y** |
| 157 | **N-(2-Fluorophenyl)-N-{4-Phenyl-1-[2-(Thiophen-2-Yl)Ethyl]Piperidin-4-Yl}Propanamide** |  | N-(2-Fluorophenyl)-N-{4-Phenyl-1-[2-(Thiophen-2-Yl)Ethyl]Piperidin-4-Yl}Propanamide | N | N | N | N | **Y** |
| 158 | **N-(2-Fluorophenyl)-N-{4-Phenyl-1-[2-(Thiophen-3-Yl)Ethyl]Piperidin-4-Yl}Propanamide** |  | N-(2-Fluorophenyl)-N-{4-Phenyl-1-[2-(Thiophen-3-Yl)Ethyl]Piperidin-4-Yl}Propanamide | N | N | N | N | **Y** |
| 159 | **N-(3-Fluorophenyl)-N-[1-(2-Hydroxy-2-Phenylethyl)-3-Methylpiperidin-4-Yl]Propanamide** |  | N-(3-Fluorophenyl)-N-[1-(2-Hydroxy-2-Phenylethyl)-3-Methylpiperidin-4-Yl]Propanamide | N | N | N | N | **Y** |
| 160 | **N-(4-Fluorophenyl)-N-[1-(2-Hydroxy-2-Phenylethyl)-3-Methylpiperidin-4-Yl]Propanamide** |  | N-(4-Fluorophenyl)-N-[1-(2-Hydroxy-2-Phenylethyl)-3-Methylpiperidin-4-Yl]Propanamide | N | N | N | N | **Y** |
| 161 | **N-[1-(2-Cyclopropyl-2-Hydroxyethyl)-3-Methylpiperidin-4-Yl]-N-Phenylpropanamide** |  | N-[1-(2-Cyclopropyl-2-Hydroxyethyl)-3-Methylpiperidin-4-Yl]-N-Phenylpropanamide | N | N | N | N | **Y** |
| 162 | **N-[1-(2-Hydroxy-2-Phenylethyl)-3-Methylpiperidin-4-Yl]-2-Methoxy-N-Phenylacetamide** |  | N-[1-(2-Hydroxy-2-Phenylethyl)-3-Methylpiperidin-4-Yl]-2-Methoxy-N-Phenylacetamide | N | N | N | N | **Y** |
| 163 | **N-[1-(2-Hydroxy-2-Phenylethyl)-3-Methylpiperidin-4-Yl]-N-(3-Methoxyphenyl)Propanamide** |  | N-[1-(2-Hydroxy-2-Phenylethyl)-3-Methylpiperidin-4-Yl]-N-(3-Methoxyphenyl)Propanamide | N | N | N | N | **Y** |
| 164 | **N-[1-(2-Hydroxy-2-Phenylethyl)-3-Methylpiperidin-4-Yl]-N-(Pyridin-2-Yl)Propanamide** |  | N-[1-(2-Hydroxy-2-Phenylethyl)-3-Methylpiperidin-4-Yl]-N-(Pyridin-2-Yl)Propanamide | N | N | N | N | **Y** |
| 165 | **N-[1-(2-Hydroxy-2-Phenylethyl)-3-Methylpiperidin-4-Yl]-N-(Pyridin-3-Yl)Propanamide** |  | N-[1-(2-Hydroxy-2-Phenylethyl)-3-Methylpiperidin-4-Yl]-N-(Pyridin-3-Yl)Propanamide | N | N | N | N | **Y** |
| 166 | **N-[1-(2-Hydroxy-2-Phenylethyl)-3-Methylpiperidin-4-Yl]-N-Phenylfuran-2-Carboxamide** |  | N-[1-(2-Hydroxy-2-Phenylethyl)-3-Methylpiperidin-4-Yl]-N-Phenylfuran-2-Carboxamide | N | N | N | N | **Y** |
| 167 | **N-[1-(2-Hydroxy-2-Phenylethyl)-3-Methylpiperidin-4-Yl]-N-Phenylfuran-3-Carboxamide** |  | N-[1-(2-Hydroxy-2-Phenylethyl)-3-Methylpiperidin-4-Yl]-N-Phenylfuran-3-Carboxamide | N | N | N | N | **Y** |
| 168 | **N-[1-(2-Hydroxy-2-Phenylethyl)-3-Methylpiperidin-4-Yl]-N-Phenylthiophene-2-Carboxamide** |  | N-[1-(2-Hydroxy-2-Phenylethyl)-3-Methylpiperidin-4-Yl]-N-Phenylthiophene-2-Carboxamide | N | N | N | N | **Y** |
| 169 | **N-[1-(2-Hydroxy-2-Phenylethyl)-3-Methylpiperidin-4-Yl]-N-Phenylthiophene-3-Carboxamide** |  | N-[1-(2-Hydroxy-2-Phenylethyl)-3-Methylpiperidin-4-Yl]-N-Phenylthiophene-3-Carboxamide | N | N | N | N | **Y** |
| 170 | **N-[1-(2-Hydroxy-2-Phenylethyl)-4- (Methoxymethyl)-3-Methylpiperidin-4-Yl]-N-Phenylpropanamide** |  | N-[1-(2-Hydroxy-2-Phenylethyl)-4- (Methoxymethyl)-3-Methylpiperidin-4-Yl]-N-Phenylpropanamide | N | N | N | N | **Y** |
| 171 | **N-[4-(4-Methyl-1,3-Thiazol-2-Yl)-1- (2-Phenylethyl)Piperidin-4-Yl]-N-Phenylpropanamide** |  | N-[4-(4-Methyl-1,3-Thiazol-2-Yl)-1- (2-Phenylethyl)Piperidin-4-Yl]-N-Phenylpropanamide | N | N | N | N | **Y** |
| 172 | **N-{1-[(2r,3r)-3-Hydroxy-1,2,3,4-Tetrahydronaphthalen-2-Yl]-3-Methylpiperidin-4-Yl}-N-Phenylpropanamide** |  | N-{1-[(2r,3r)-3-Hydroxy-1,2,3,4-Tetrahydronaphthalen-2-Yl]-3-Methylpiperidin-4-Yl}-N-Phenylpropanamide | N | N | N | N | **Y** |
| 173 | **N-{1-[2-(3,5-Dimethyl-1h-Pyrazol-1-Yl)Ethyl]-4-Phenylpiperidin-4-Yl}-N- (2-Fluorophenyl)Propanamide** |  | N-{1-[2-(3,5-Dimethyl-1h-Pyrazol-1-Yl)Ethyl]-4-Phenylpiperidin-4-Yl}-N- (2-Fluorophenyl)Propanamide | N | N | N | N | **Y** |
| 174 | **N-{1-[2-(4-Ethyl-5-Oxo-4,5-Dihydro-1h-Tetrazol-1-Yl)Ethyl]-4- (1,3-Thiazol-2-Yl)Piperidin-4-Yl}-N- (2-Fluorophenyl)Propanamide** |  | N-{1-[2-(4-Ethyl-5-Oxo-4,5-Dihydro-1h-Tetrazol-1-Yl)Ethyl]-4- (1,3-Thiazol-2-Yl)Piperidin-4-Yl}-N- (2-Fluorophenyl)Propanamide | N | N | N | N | **Y** |
| 175 | **N-{1-[2-(Furan-2-Yl)-2-Hydroxyethyl]-4- (Methoxymethyl)Piperidin-4-Yl}-N-Phenylpropanamide** |  | N-{1-[2-(Furan-2-Yl)-2-Hydroxyethyl]-4- (Methoxymethyl)Piperidin-4-Yl}-N-Phenylpropanamide | N | N | N | N | **Y** |
| 176 | **N-{1-[2-Hydroxy-2-(1-Methyl-1h-Pyrrol-2-Yl)Ethyl]-3-Methylpiperidin-4-Yl}-N-Phenylpropanamide** |  | N-{1-[2-Hydroxy-2-(1-Methyl-1h-Pyrrol-2-Yl)Ethyl]-3-Methylpiperidin-4-Yl}-N-Phenylpropanamide | N | N | N | N | **Y** |
| 177 | **N-{1-[2-Hydroxy-2-(Pyridin-3-Yl)Ethyl]-3-Methylpiperidin-4-Yl}-N-Phenylpropanamide** |  | N-{1-[2-Hydroxy-2-(Pyridin-3-Yl)Ethyl]-3-Methylpiperidin-4-Yl}-N-Phenylpropanamide | N | N | N | N | **Y** |
| 178 | **N-{1-[2-Hydroxy-2-(Pyridin-4-Yl)Ethyl]-3-Methylpiperidin-4-Yl}-N-Phenylpropanamide** |  | N-{1-[2-Hydroxy-2-(Pyridin-4-Yl)Ethyl]-3-Methylpiperidin-4-Yl}-N-Phenylpropanamide | N | N | N | N | **Y** |
| 179 | **N-{1-[2-Hydroxy-2-(Thiophen-2-Yl)Ethyl]-3-Methylpiperidin-4-Yl}-N-(3-Methoxyphenyl)Propanamide** |  | N-{1-[2-Hydroxy-2-(Thiophen-2-Yl)Ethyl]-3-Methylpiperidin-4-Yl}-N-(3-Methoxyphenyl)Propanamide | N | N | N | N | **Y** |
| 180 | **N-{3,5-Dimethyl-1-[2-(1h-Pyrazol-1-Yl)Ethyl]Piperidin-4-Yl}-2-Methoxy-N-Phenylacetamide** |  | N-{3,5-Dimethyl-1-[2-(1h-Pyrazol-1-Yl)Ethyl]Piperidin-4-Yl}-2-Methoxy-N-Phenylacetamide | N | N | N | N | **Y** |
| 181 | **N-Adamantyl-Fentanyl** |  | N-(Adamantan-1-yl)-N-[1-(2-phenylethyl)piperidin-4-yl]propanamide | N | N | N | N | **Y** |
| 182 | **N-Benzoxazolyl-Fentanyl** |  | N-(1,3-Benzoxazol-6-yl)-N-[1-(2-phenylethyl)piperidin-4-yl]propanamide | N | N | N | N | **Y** |
| 183 | **N-Benzyl-Acetylfentanyl** |  | N-(1-Benzylpiperidin-4-yl)-N-phenylacetamide | N | N | N | N | **Y** |
| 184 | **N-Benzyl-Butyrylfentanyl** |  | N-(1-Benzylpiperidin-4-yl)-N-phenylbutanamide | N | N | N | N | **Y** |
| 185 | **N-Benzylcarfentanil** | Benzylcarfentanil | Methyl 1-benzyl-4-[phenyl(propanoyl)amino]piperidine-4-carboxylate | N | N | N | N | **Y** |
| 186 | **N-Benzyl-P-Fluoro-Isobutyrylfentanyl** |  | 1-(1-Benzylpiperidin-4-yl)-1-(4-fluorophenyl)-3-methylbutan-2-one | N | N | N | N | **Y** |
| 187 | **N-Furanylethylfentanyl** |  | N-{1-[2-(Furan-2-yl)ethyl]piperidin-4-yl}-N-phenylpropanamide | N | N | N | N | **Y** |
| 188 | **N-Methyl-Acetylfentanyl** | N-Methyl-acetyl-norfentanyl | N-(1-Methylpiperidin-4-yl)-N-phenylacetamide | N | N | N | N | **Y** |
| 189 | **N-Methyl-Butyrylfentanyl** |  | N-(1-Methylpiperidin-4-yl)-N-phenylbutanamide | N | N | N | N | **Y** |
| 190 | **N-Phenyl-N-[1-(2-Phenylethyl)-4- (1,3-Thiazol-2-Yl)Piperidin-4-Yl]Propanamide** |  | N-Phenyl-N-[1-(2-phenylethyl)-4-(1,3-thiazol-2-yl)piperidin-4-yl]propanamide | N | N | N | N | **Y** |
| 191 | **N-Phenyl-N-{4-Phenyl-1-[2-(1h-Pyrazol-1-Yl)Ethyl]Piperidin-4-Yl}Propanamide** |  | N-Phenyl-N-{4-Phenyl-1-[2-(1h-Pyrazol-1-Yl)Ethyl]Piperidin-4-Yl}Propanamide | N | N | N | N | **Y** |
| 192 | **N-Phenyl-N-{4-Phenyl-1-[2-(Pyridin-2-Yl)Ethyl]Piperidin-4-Yl}Propanamide** |  | N-Phenyl-N-{4-Phenyl-1-[2-(Pyridin-2-Yl)Ethyl]Piperidin-4-Yl}Propanamide | N | N | N | N | **Y** |
| 193 | **N-Phenyl-N-{4-Phenyl-1-[2-(Thiophen-2-Yl)Ethyl]Piperidin-4-Yl}Propanamide** |  | N-Phenyl-N-{4-Phenyl-1-[2-(Thiophen-2-Yl)Ethyl]Piperidin-4-Yl}Propanamide | N | N | N | N | **Y** |
| 194 | **N-Phenyl-N-{4-Phenyl-1-[2-(Thiophen-3-Yl)Ethyl]Piperidin-4-Yl}Propanamide** |  | N-Phenyl-N-{4-Phenyl-1-[2-(Thiophen-3-Yl)Ethyl]Piperidin-4-Yl}Propanamide | N | N | N | N | **Y** |
| 195 | **N-Quinolinyl-Fentanyl** |  | N-[1-(2-Phenylethyl)piperidin-4-yl]-N-(quinolin-8-yl)propanamide | N | N | N | N | **Y** |
| 196 | **O-Fluoro-Despropionoylfentanyl** | 2′-Fluoro-despropionoylfentanyl | N-(2-Fluorophenyl)-1-(2-phenylethyl)piperidin-4-amine | N | N | N | N | **Y** |
| 197 | **O-Methyl-Benzoylfentanyl** | 2′-Methyl-benzoylfentanyl | N-(2-Methylphenyl)-N-[1-(2-phenylethyl)piperidin-4-yl]benzamide | N | N | N | N | **Y** |
| 198 | **O-Methyl-Cyclopropylfentanyl** | 2′-Methyl-cyclopropylfentanyl | N-(2-Methylphenyl)-N-[1-(2-phenylethyl)piperidin-4-yl]cyclopropanecarboxamide | N | N | N | N | **Y** |
| 199 | **O-Methylfentanyl** | 2′-Methylfentanyl | N-(2-Methylphenyl)-N-[1-(2-phenylethyl)piperidin-4-yl]propanamide | N | N | N | N | **Y** |
| 200 | **Ortho-Methyl Furanyl Fentanyl** | o-Methyl-furanylfentanyl; 2′-Methyl-furanylfentanyl; 2′-Me-Fu-F | N-(2-Methylphenyl)-N-[1-(2-phenylethyl)piperidin-4-yl]furan-2-carboxamide | N | N | N | N | **Y** |
| 201 | **Para-Chlorofuranylfentanyl** | p-Chloro-furanylfentanyl; 4′-Chloro-furanylfentanyl; p-Cl-Fu-F | N-(4-Chlorophenyl)-N-[1-(2-phenylethyl)piperidin-4-yl]furan-2-carboxamide | N | N | N | N | **Y** |
| 202 | **P-Bromofentanyl** | p-Bromofentanyl; 4′-Bromofentanyl | N-(4-Bromophenyl)-N-[1-(2-phenylethyl)piperidin-4-yl]propanamide | N | N | N | N | **Y** |
| 203 | **P-Fluoro-Crotonylfentanyl** | p-Fluoro-crotonylfentanyl; 4′-Fluoro-crotonylfentanyl | (2E)-N-(4-Fluorophenyl)-N-[1-(2-phenylethyl)piperidin-4-yl]but-2-enamide | N | N | N | N | **Y** |
| 204 | **P-Fluoro-Cyclopentylfentanyl** | p-Fluoro-cyclopentylfentanyl; 4′-Fluoro-cyclopentylfentanyl | N-(4-Fluorophenyl)-N-[1-(2-phenylethyl)piperidin-4-yl]cyclopentanecarboxamide | N | N | N | N | **Y** |
| 205 | **P-Fluoro-Cyclopropylfentanyl** | p-Fluoro-cyclopropylfentanyl; 4′-Fluoro-cyclopropylfentanyl | N-(4-Fluorophenyl)-N-[1-(2-phenylethyl)piperidin-4-yl]cyclopropanecarboxamide | N | N | N | N | **Y** |
| 206 | **P-Fluoro-Furan-3-Ylfentanyl** | p-Fluoro-furan-3-ylfentanyl; 4′-Fluoro-furan-3-ylfentanyl | N-(4-Fluorophenyl)-N-[1-(2-phenylethyl)piperidin-4-yl]furan-3-carboxamide | N | N | N | N | **Y** |
| 207 | **P-Fluoro-Furanylethylfentanyl** | p-Fluoro-furanylethylfentanyl; 4′-Fluoro-furanylethylfentanyl | 1-(4-Fluorophenyl)-1-{1-[2-(furan-2-yl)ethyl]piperidin-4-yl}butan-2-one | N | N | N | N | **Y** |
| 208 | **P-Fluoro-Furanylfentanyl** | p-Fluoro-furanylfentanyl; 4′-Fluoro-furanylfentanyl; p-F-Fu-F | N-(4-Fluorophenyl)-N-[1-(2-phenylethyl)piperidin-4-yl]furan-2-carboxamide | N | N | N | N | **Y** |
| 209 | **P-Fluoro-Furanylremifentanil** | p-Fluoro-furanylremifentanil | Methyl 4-[(4-fluorophenyl)(furan-2-carbonyl)amino]-1-(3-methoxy-3-oxopropyl)piperidine-4-carboxylate | N | N | N | N | **Y** |
| 210 | **P-Fluoro-Methoxyacetylfentanyl** | p-Fluoro-methoxyacetylfentanyl; 4′-Fluoro-methoxyacetylfentanyl | N-(4-Fluorophenyl)-2-methoxy-N-[1-(2-phenylethyl)piperidin-4-yl]acetamide | N | N | N | N | **Y** |
| 211 | **P-Fluoro-Tetrahydrofuranylfentanyl** | p-Fluoro-tetrahydrofuranylfentanyl; 4′-Fluoro-tetrahydrofuranylfentanyl | N-(4-Fluorophenyl)-N-[1-(2-phenylethyl)piperidin-4-yl]oxolane-2-carboxamide | N | N | N | N | **Y** |
| 212 | **P-Fluoro-Thiofentanyl** | p-Fluoro-thiofentanyl; 4′-Fluoro-thiofentanyl | N-(4-Fluorophenyl)-N-{1-[2-(thiophen-2-yl)ethyl]piperidin-4-yl}propanamide | N | N | N | N | **Y** |
| 213 | **Pharaohfentanyl** |  | N-[1-(2-Hydroxy-2-phenylethyl)-4-methylpiperidin-4-yl]-N-phenylpropanamide | N | N | N | N | **Y** |
| 214 | **Phenaridine** | 2,5-Dimethylfentanyl | N-[2,5-Dimethyl-1-(2-phenylethyl)piperidin-4-yl]-N-phenylpropanamide | N | N | N | N | **Y** |
| 215 | **Phenoxyethyl-Norfentanyl** | POE-F | N-[1-(2-Phenoxyethyl)piperidin-4-yl]-N-phenylpropanamide | N | N | N | N | **Y** |
| 216 | **Phenylacetylfentanyl** |  | N,2-Diphenyl-N-[1-(2-phenylethyl)piperidin-4-yl]acetamide | N | N | N | N | **Y** |
| 217 | **Phenylpropyl-Norfentanyl** | PP-F | N-Phenyl-N-[1-(3-phenylpropyl)piperidin-4-yl]propanamide | N | N | N | N | **Y** |
| 218 | **P-Iodofentanyl** | p-Iodofentanyl; 4′-Iodofentanyl | N-(4-Iodophenyl)-N-[1-(2-phenylethyl)piperidin-4-yl]propanamide | N | N | N | N | **Y** |
| 219 | **Pivaloylfentanyl** |  | 2,2-Dimethyl-N-phenyl-N-[1-(2-phenylethyl)piperidin-4-yl]propanamide | N | N | N | N | **Y** |
| 220 | **P-Methoxy-Acetylfentanyl** | p-Methoxy-acetylfentanyl; 4′-Methoxy-acetylfentanyl; 4′-MeO-AF | N-(4-Methoxyphenyl)-N-[1-(2-phenylethyl)piperidin-4-yl]acetamide | N | N | N | N | **Y** |
| 221 | **P-Methoxy-Butyrylfentanyl** | p-Methoxy-butyrylfentanyl; 4′-Methoxy-butyrylfentanyl; p-MeO-BF | N-(4-Methoxyphenyl)-N-[1-(2-phenylethyl)piperidin-4-yl]butanamide | N | N | N | N | **Y** |
| 222 | **P-Methoxy-Methoxyacetylfentanyl** | p-Methoxy-methoxyacetylfentanyl; 4′-Methoxy-methoxyacetylfentanyl | 2-Methoxy-N-(4-methoxyphenyl)-N-[1-(2-phenylethyl)piperidin-4-yl]acetamide | N | N | N | N | **Y** |
| 223 | **P-Methoxy-Tetrahydrofuranylfentanyl** | p-Methoxy-tetrahydrofuranylfentanyl; 4′-Methoxy-tetrahydrofuranylfentanyl | N-(4-Methoxyphenyl)-N-[1-(2-phenylethyl)piperidin-4-yl]oxolane-2-carboxamide | N | N | N | N | **Y** |
| 224 | **P-Methoxy-Valerylfentanyl** | 4′-Methoxy-tetrahydrofuranylfentanyl | N-(4-Methoxyphenyl)-N-[1-(2-phenylethyl)piperidin-4-yl]oxolane-2-carboxamid | N | N | N | N | **Y** |
| 225 | **P-Methyl-Acetylfentanyl** | 4′-Methyl-acetylfentanyl | N-(4-Methylphenyl)-N-[1-(2-phenylethyl)piperidin-4-yl]acetamide | N | N | N | N | **Y** |
| 226 | **P-Methyl-Cyclopropylfentanyl** | 4′-Methyl-cyclopropylfentanyl | N-(4-Methylphenyl)-N-[1-(2-phenylethyl)piperidin-4-yl]cyclopropanecarboxamide | N | N | N | N | **Y** |
| 227 | **Propylnorfentanyl** |  | N-Phenyl-N-(1-propylpiperidin-4-yl)propanamide | N | N | N | N | **Y** |
| 228 | **Psicofentanil** | EAZ-91-05 | 1-Azabicyclo[2.2.2]octan-3-yl 1-[2-(1H-indol-3-yl)ethyl]-4-[phenyl(propanoyl)amino]piperidine-4-carboxylate | N | N | N | N | **Y** |
| 229 | **P-TFM-Fentanyl** | 4′-TFM-fentanyl | N-[1-(2-Phenylethyl)piperidin-4-yl]-N-[4-(trifluoromethyl)phenyl]propanamide | N | N | N | N | **Y** |
| 230 | **Remifentanil Bis Ethyl Ester** |  | Ethyl 1-(3-ethoxy-3-oxopropyl)-4-[phenyl(propanoyl)amino]piperidine-4-carboxylate | N | N | N | N | **Y** |
| 231 | **Tetrahydrofuran-Fentanyl** | tetrahydrofuranfentanyl; THF-F (hydrochloride) | N-(1-phenethylpiperidin-4-yl)-N-phenyltetrahydrofuran-2-carboxamide, monohydrochloride | N | N | N | N | **Y** |
| 232 | **Thiafentanil** | A-3080 | Methyl 4-[(methoxyacetyl)(phenyl)amino]-1-[2-(thiophen-2-yl)ethyl]piperidine-4-carboxylate | N | N | N | N | **Y** |
| 233 | **Thiophenoylfentanyl** |  | N-Phenyl-N-[1-(2-phenylethyl)piperidin-4-yl]thiophene-2-carboxamide | N | N | N | N | **Y** |
| 234 | **Α-Methyl-Isobutyrylfentanyl** | α-Methyl-isobutyrylfentanyl | 2-Methyl-N-phenyl-N-[1-(1-phenylpropan-2-yl)piperidin-4-yl]propanamide | N | N | N | N | **Y** |

*Table S1 –* *NPS.Finder® fentanyl analogues; IUPAC names and comparisons between the different databases*
