# Supplementary material for: Novel Opioids: Systematic Web Crawling Within the e-Psychonauts’ Scenario
Source: Front Neurosci. 2020 Mar 18;14:149. doi: 10.3389/fnins.2020.00149 (PMC7093327; doi:10.3389/fnins.2020.00149)
Supplement: Supplementary file 2 [file Table_2.docx]

| **N** | ***NPS Finder name*** | ***other names*** | ***IUPAC*** | ***INCB Yellow list (March 2019)*** | ***EDND (April 2019)*** | ***UNODC EWA NPS (July 2019)*** | ***only in NPS Finder*** | ***brief description*** | ***ATC, prescribed/OTC, withdrawn, metabolite, experimental*** |
| --- | --- | --- | --- | --- | --- | --- | --- | --- | --- |
| 1 | acetyldihydrocodeine | Dihydrocodeine 6-acetate; Dihydrothebacone | [(4R,4aR,7S,7aR,12bS)-9-methoxy-3-methyl-2,4,4a,5,6,7,7a,13-octahydro-1H-4,12-methanobenzofuro[3,2-e]isoquinolin-7-yl] acetate | Y | N | N | N | Dihydrocodeine derivative | R05DA12 |
| 2 | alfentanil | R-39209; Alfenta; Rapifen | N-[1-[2-(4-ethyl-5-oxotetrazol-1-yl)ethyl]-4-(methoxymethyl)piperidin-4-yl]-N-phenylpropanamide | Y | N | N | N | Anilidopiperidines | N01AH02 |
| 3 | anileridine | Leritine | ethyl 1-[2-(4-aminophenyl)ethyl]-4-phenylpiperidine-4-carboxylate | Y | N | N | N | 4-phenylpiperidines, pethidines (meperidines) | N01AH05 |
| 4 | bezitramide | Benzitramide; Burgodin | 4-[4-(2-oxo-3-propanoylbenzimidazol-1-yl)piperidin-1-yl]-2,2-diphenylbutanenitrile | Y | N | N | N | Pirinitramides, prodrug, marketed | N02AC05 |
| 5 | buprenorphine | Subutex | (1S,2S,6R,14R,15R,16R)-5-(cyclopropylmethyl)-16-[(2S)-2-hydroxy-3,3-dimethylbutan-2-yl]-15-methoxy-13-oxa-5-azahexacyclo[13.2.2.12,8.01,6.02,14.012,20]icosa-8(20),9,11-trien-11-ol | N | N | N | N | Oripavine derivative | N02AE01; N07BC01 |
| 6 | butorphanol | Butorfanol; Stadol | (1S,9R,10S)-17-(cyclobutylmethyl)-17-azatetracyclo[7.5.3.01,10.02,7]heptadeca-2(7),3,5-triene-4,10-diol | N | N | N | N | Morphinan, others | N02AF01 |
| 7 | codeine | 3-Methoxymorphine | (4R,4aR,7S,7aR,12bS)-9-methoxy-3-methyl-2,4,4a,7,7a,13-hexahydro-1H-4,12-methanobenzofuro[3,2-e]isoquinolin-7-ol | Y | N | N | N | Opium and poppy straw derivatives, opium alkaloids | R05DA04 |
| 8 | Co-Proxamol | Distalgesic; Coproxamol |  | Y (see dextropropoxyphene) | N | N | N | Acetaminophen and dextropropoxyphene drug combination | N02AC54 |
| 9 | dextromethorphan | d-Methorphan; DXM; DM | (1S,9S,10S)-4-methoxy-17-methyl-17-azatetracyclo[7.5.3.01,10.02,7]heptadeca-2(7),3,5-triene | Dextromethorphan and dextrorphan are not under international control. | N | N | N | Morphinans, morphinan series | R05DA09 |
| 10 | dextromoramide | Palfium; Palphium; Jetrium; Dimorlin | (3S)-3-methyl-4-morpholin-4-yl-2,2-diphenyl-1-pyrrolidin-1-ylbutan-1-one | Y | N | N | N | Open chain | N02AC01 |
| 11 | dextropropoxyphene | propoxyphene; Darvon | [(2S,3R)-4-(dimethylamino)-3-methyl-1,2-diphenylbutan-2-yl] propanoate | Y | N | N | N | Open chain | N02AC04 |
| 12 | diamorphine | Diamorphine hydrochloride (approved); diamorphine; diacetylmorphine; morphine diacetate; dope; H; smack; junk; horse; brown | [(4R,4aR,7S,7aR,12bS)-9-acetyloxy-3-methyl-2,4,4a,7,7a,13-hexahydro-1H-4,12-methanobenzofuro[3,2-e]isoquinolin-7-yl] acetate | Y | N | N | N | Morphine derivative, 3,6-diesters of morphine | N07BC06 |
| 13 | difenoxin | Motofen; R-15403 | 1-(3-cyano-3,3-diphenylpropyl)-4-phenylpiperidine-4-carboxylic acid | Y | N | N | N | 4-phenylpiperidines, pethidines (meperidines), diphenylpropylamine derivatives | A07DA04 |
| 14 | dihydrocodeine | Hydrocodeine; 6-alpha-Hydrocodol | (4R,4aR,7S,7aR,12bS)-9-methoxy-3-methyl-2,4,4a,5,6,7,7a,13-octahydro-1H-4,12-methanobenzofuro[3,2-e]isoquinolin-7-ol | Y | N | N | N | Dihydrocodeine series | N02AA08 |
| 15 | diphenoxylate | R-1132 | ethyl 1-(3-cyano-3,3-diphenylpropyl)-4-phenylpiperidine-4-carboxylate | Y | N | N | N | 4-phenylpiperidines, pethidines (meperidines), diphenylpropylamine derivatives | A07DA01 |
| 16 | eluxadoline | Viberzi; Truberzi | 5-[[[(2S)-2-amino-3-(4-carbamoyl-2,6-dimethylphenyl)propanoyl]-[(1S)-1-(5-phenyl-1H-imidazol-2-yl)ethyl]amino]methyl]-2-methoxybenzoic acid | N | N | N | N | others | A07DA06 |
| 17 | ethylmorphine | codethyline; dionine; ethyl morphine | (4R,4aR,7S,7aR,12bS)-9-ethoxy-3-methyl-2,4,4a,7,7a,13-hexahydro-1H-4,12-methanobenzofuro[3,2-e]isoquinolin-7-ol | Y | N | N | N | Codeine-dionine family | R05DA01 |
| 18 | fentanyl | fentanil; Actiq; Duragesic; Fentora | N-phenyl-N-[1-(2-phenylethyl)piperidin-4-yl]propanamide | Y | N | N | N | Anilidopiperidines | N01AH01; N02AB03 |
| 19 | hydrocodone | Dihydrocodeinone; Vicodin; Norco | (4R,4aR,7aR,12bS)-9-methoxy-3-methyl-1,2,4,4a,5,6,7a,13-octahydro-4,12-methanobenzofuro[3,2-e]isoquinolin-7-one | Y | N | N | N | Morphinones and morphols | R05DA03 |
| 20 | hydromorphone | dihydromorphinone; Dilaudid | (4R,4aR,7aR,12bS)-9-hydroxy-3-methyl-1,2,4,4a,5,6,7a,13-octahydro-4,12-methanobenzofuro[3,2-e]isoquinolin-7-one | Y | N | N | N | Morphinones and morphols | N02AA03 |
| 21 | ketobemidone | Cliradon; Cymidon; Ketogan; Ketorax | 1-[4-(3-hydroxyphenyl)-1-methylpiperidin-4-yl]propan-1-one | Y | N | N | N | 4-phenylpiperidines, ketobemidones | N02AB01 |
| 22 | levacetylmethadol | levomethadyl acetate; levo-α-acetylmethadol; LAAM; OrLAAM | [(3S,6S)-6-(dimethylamino)-4,4-diphenylheptan-3-yl] acetate | N | N | N | N | Open chain, methadone related | N07BC03 |
| 23 | loperamide | Imodium | 4-[4-(4-chlorophenyl)-4-hydroxypiperidin-1-yl]-N,N-dimethyl-2,2-diphenylbutanamide | N | N | N | N | 4-phenylpiperidines, others | A07DA03 |
| 24 | methadone | Dolophine; Amidone; Biodone; Physeptone; Adanon; Diaminon; Ketalgin | 6-(dimethylamino)-4,4-diphenylheptan-3-one | Y | N | N | N | Open chain | N07BC02 |
| 25 | morphine | Statex; MSContin; Oramorph; Sevredol | (4R,4aR,7S,7aR,12bS)-3-methyl-2,4,4a,7,7a,13-hexahydro-1H-4,12-methanobenzofuro[3,2-e]isoquinoline-7,9-diol | Y | N | N | N | Opium and poppy straw derivatives, opium alkaloids | N02AA01 |
| 26 | nalbuphine | Nubain | (4R,4aS,7S,7aR,12bS)-3-(cyclobutylmethyl)-1,2,4,5,6,7,7a,13-octahydro-4,12-methanobenzofuro[3,2-e]isoquinoline-4a,7,9-triol | N | N | N | N | Morphinan, others | N02AF02 |
| 27 | nalmefene | nalmetrene; Selincro | (4R,4aS,7aS,12bS)-3-(cyclopropylmethyl)-7-methylidene-2,4,5,6,7a,13-hexahydro-1H-4,12-methanobenzofuro[3,2-e]isoquinoline-4a,9-diol | N | N | N | N | Opioid antagonists and inverse agonists | N07BB05 |
| 28 | nalorphine | Allorphine; N-allylnormorphine; Lethidrone; Nalline | (4R,4aR,7S,7aR,12bS)-3-prop-2-enyl-2,4,4a,7,7a,13-hexahydro-1H-4,12-methanobenzofuro[3,2-e]isoquinoline-7,9-diol | N | N | N | N | Opioid antagonists and inverse agonists, mixed opioid agonist–antagonist | V03AB02 |
| 29 | naloxegol | PEGylated naloxol; Movantik; Moventig; NKTR-118 | (4R,4aS,7S,7aR,12bS)-7-[2-[2-[2-[2-[2-[2-(2-methoxyethoxy)ethoxy]ethoxy]ethoxy]ethoxy]ethoxy]ethoxy]-3-prop-2-enyl-1,2,4,5,6,7,7a,13-octahydro-4,12-methanobenzofuro[3,2-e]isoquinoline-4a,9-diol | N | N | N | N | Opioid antagonists and inverse agonists, opioid antagonist, α-naloxol antagonist | A06AH03 |
| 30 | naloxone | Narcan; Evzio | (4R,4aS,7aR,12bS)-4a,9-dihydroxy-3-prop-2-enyl-2,4,5,6,7a,13-hexahydro-1H-4,12-methanobenzofuro[3,2-e]isoquinolin-7-one | N | N | N | N | Opioid antagonists and inverse agonists, opioid antagonist | A06AH04; V03AB15 |
| 31 | naltrexone | ReVia; Vivitrol | (4R,4aS,7aR,12bS)-3-(cyclopropylmethyl)-4a,9-dihydroxy-2,4,5,6,7a,13-hexahydro-1H-4,12-methanobenzofuro[3,2-e]isoquinolin-7-one | N | N | N | N | Opioid antagonists and inverse agonists, opioid antagonist | N07BB04 |
| 32 | nicomorphine | Vilan; Subellan; Gevilan; MorZet | [(4R,4aR,7S,7aR,12bS)-3-methyl-9-(pyridine-3-carbonyloxy)-2,4,4a,7,7a,13-hexahydro-1H-4,12-methanobenzofuro[3,2-e]isoquinolin-7-yl] pyridine-3-carboxylate | Y | N | N | N | Ester of morphine | N02AA04 |
| 33 | normethadone | Phenyldimazone; desmethylmethadone; Cophylac; Dacartil; Eucopon; Mepidon; Noramidone; Normedon | 6-(dimethylamino)-4,4-diphenylhexan-3-one | Y | N | N | N | Open chain | R05DA06 |
| 34 | noscapine | Narcotine; Nectodon; Nospen; Anarcotine; Opiane | (3S)-6,7-dimethoxy-3-[(5R)-4-methoxy-6-methyl-7,8-dihydro-5H-[1,3]dioxolo[4,5-g]isoquinolin-5-yl]-3H-2-benzofuran-1-one | N | N | N | N | Opium alkaloid | R05DA07 |
| 35 | opium | Lachryma papaveris; poppy tears |  | Y | N | N | N | Opium and poppy straw derivatives, crude opiate extracts whole opium products | N02AA02; A07DA02; |
| 36 | oxycodone | dihydrohydroxycodeinone; OxyContin; Eukodal; eucodal | (4R,4aS,7aR,12bS)-4a-hydroxy-9-methoxy-3-methyl-2,4,5,6,7a,13-hexahydro-1H-4,12-methanobenzofuro[3,2-e]isoquinolin-7-one | Y | N | N | N | Morphinones and morphols | N02AA05 |
| 37 | pentazocine | Talwin | (1R,9R,13R)-1,13-dimethyl-10-(3-methylbut-2-enyl)-10-azatricyclo[7.3.1.02,7]trideca-2(7),3,5-trien-4-ol | N | N | N | N | Benzomorphan derivatives, agonist/antagonist | N02AD01 |
| 38 | pethidine | meperidine; Demerol | ethyl 1-methyl-4-phenylpiperidine-4-carboxylate | Y | N | N | N | 4-phenylpiperidines, pethidines (meperidines) | N02AB02 |
| 39 | phenazocine | Prinadol; Narphen | 1,13-dimethyl-10-(2-phenylethyl)-10-azatricyclo[7.3.1.02,7]trideca-2(7),3,5-trien-4-ol | Y | N | N | N | Benzomorphan derivatives | N02AD02 |
| 40 | phenoperidine | Operidine; Lealgin | ethyl 1-(3-hydroxy-3-phenylpropyl)-4-phenylpiperidine-4-carboxylate | Y | N | N | N | 4-phenylpiperidines, pethidines (meperidines) | N01AH04 |
| 41 | pholcodine | Logicin | (4R,4aR,7S,7aR,12bS)-3-methyl-9-(2-morpholin-4-ylethoxy)-2,4,4a,7,7a,13-hexahydro-1H-4,12-methanobenzofuro[3,2-e]isoquinolin-7-ol | Y | N | N | N | Codeine-dionine family | R05DA08 |
| 42 | piritramide | R-3365; Dipidolor; Piridolan; Pirium | 1-(3-cyano-3,3-diphenylpropyl)-4-piperidin-1-ylpiperidine-4-carboxamide | Y | N | N | N | Pirinitramides | N02AC03 |
| 43 | remifentanil | Remifentanyl; Ultiva | methyl 1-(3-methoxy-3-oxopropyl)-4-(N-propanoylanilino)piperidine-4-carboxylate | Y | N | N | N | Anilidopiperidines | N01AH06 |
| 44 | sufentanil | Dsuvia; Sufenta | N-[4-(methoxymethyl)-1-(2-thiophen-2-ylethyl)piperidin-4-yl]-N-phenylpropanamide | Y | N | N | N | Anilidopiperidines | N01AH03 |
| 45 | tapentadol | Nucynta; Palexia; Yantil; Tapenta; Tapal | 3-[(2R,3R)-1-(dimethylamino)-2-methylpentan-3-yl]phenol | N | N | N | N | others | N02AX06 |
| 46 | thebacon | tebacon; Acedicon; Diacodin dihydrocodeinone enol acetate | (9-methoxy-3-methyl-2,4,4a,5,7a,13-hexahydro-1H-4,12-methanobenzofuro[3,2-e]isoquinolin-7-yl) acetate | Y | N | N | N | Codeine-dionine family, derivative of acetyldihydrocodeine | R05DA10 |
| 47 | tilidine | tilidate; Tilidin; Valoron; Valtran; dextilidine | ethyl (1S,2R)-2-(dimethylamino)-1-phenylcyclohex-3-ene-1-carboxylate | Y | N | N | N | others | N02AX01 |
| 48 | tramadol | Ultram; Zytram | (1R,2R)-2-[(dimethylamino)methyl]-1-(3-methoxyphenyl)cyclohexan-1-ol | N | N | N | N | others | N02AX02 |

*Table S2A – NPS.Finder® ATC/DDD non fentanyl analogues (ATC/DDD opioids) and comparison between the different databases.*

| **N** | ***NPS FINDER name (non-fentanyl analogs)*** | ***other names*** | **IUPAC Pubchem** | **INCB Yellow list (March 2019)** | **EDND (April 2019)** | **UNODC EWA NPS (July 2019)** | ***only in NPS Finder*** | ***brief description*** |
| --- | --- | --- | --- | --- | --- | --- | --- | --- |
| 1 | **Concentrate Of Poppy Straw** | opium straw; mowed opium straw; crushed poppy capsule; poppy chaff; poppy husk | Structure not available | Y | N | N | N | Opium and poppy straw derivatives, crude opiate extracts whole opium products |
| 2 | **Granulate opium** |  | Structure not available | Y | N | N | N | Opium and poppy straw derivatives |
| 3 | **Kratom** | Mitragyna speciosa; Ketum; Kakuam; Ithang; Thom; krath`m (Thai); ketum; kratum | Structure not available | N | N | N | N (natural occurring) | Plant |
| 4 | **Medicinal opium** |  | Structure not available | Y | N | N | N | Opium and poppy straw derivatives |
| 5 | **Papaver somniferum** | Opium; Plant of Joy; Mawseed; Joy Plant; Pen Yan; Paregoric | Structure not available | N | N | N | N (natural occurring) | Opium and poppy straw derivatives |
| 6 | **PST** | poppy-seed-tea; poppy-tea | Structure not available | Y | N | N | N | Opium and poppy straw derivatives, crude opiate extracts whole opium products |
| 7 | **Salvia Divinorum** | Ska Pastora; Shepherdess's Herb; ska Maria Pastora; yerba de Maria; Sally-D; Ska María Pastora; Seer's Sage; diviners mint; diviners sage | Structure not available | N | N | N | N (natural occurring) | Plant |
| 8 | **Tincture of opium** | Laudanum; Paregoric | Structure not available | Y | N | N | N | Opium and poppy straw derivatives, crude opiate extracts whole opium products |

*Table S2B – NPS.Finder® plants and derivatives and comparison between the different databases.*

| **N** | ***NPS FINDER name (non-fentanyl analogs)*** | ***other names*** | **IUPAC Pubchem** | **INCB Yellow list (March 2019)** | **EDND (April 2019)** | **UNODC EWA NPS (July 2019)** | ***only in NPS Finder*** | ***brief description*** | ***ATC, prescribed/OTC, withdrawn, metabolite, experimental*** |
| --- | --- | --- | --- | --- | --- | --- | --- | --- | --- |
| 1 | **03-monoacetylmorphine oxalate** |  | 3-Monoacetylmorphine: [(4R,7S,12bS)-7-hydroxy-3-methyl-2,4,4a,7,7a,13-hexahydro-1H-4,12-methanobenzofuro[3,2-e]isoquinolin-9-yl] acetate | N | N | N | N (Misuse of DrugsAct 1971 and the Misuse of Drugs Regulations 2001, UK) | Morphine derivative, active opiate metabolites | apparently not prescribed currently/in the past; morphine metabolite/intemediate (active) |
| 2 | **14-hydroxymorphine** | RAM-371 | (4R,4aS,7S,7aR,12bS)-3-methyl-1,2,4,7,7a,13-hexahydro-4,12-methanobenzofuro[3,2-e]isoquinoline-4a,7,9-triol | N | N | N | N (Misuse of DrugsAct 1971 and the Misuse of Drugs Regulations 2001, UK) | Morphine derivative | apparently not prescribed currently/in the past; morphine metabolite/intemediate |
| 3 | **1-phenethyl-4-hydroxypiperidine** |  | 1-(2-phenylethyl)piperidin-4-ol | N | N | N | **Y** | closely related to the N-phenethyl-4-piperidinone (NPP) - a precursor used for the synthesis of fentanyl | not prescribed currently/in the past; NPP related; not really new, notified for the first time in 2016 in EU |
| 4 | **2-ethyl-AP-237** | 2-methyl-bucinnazine | 1-[2-methyl-4-(3-phenylprop-2-en-1-yl)piperazin-1-yl]butan-1-one | N | Y | N | N | others | apparently not prescribed currently/in the past; bucinnazine/AP-237 derivative |
| 5 | **3-(O-carboxymethyl)morphine** |  | 2-[[(4R,4aR,7S,7aR,12bS)-7-hydroxy-3-methyl-2,4,4a,7,7a,13-hexahydro-1H-4,12-methanobenzofuro[3,2-e]isoquinolin-9-yl]oxy]acetic acid (carboxymethyl morphine) | N | N | N | N (Misuse of DrugsAct 1971 and the Misuse of Drugs Regulations 2001, UK) | Morphine derivative | apparently not prescribed currently/in the past; morphine vaccine model |
| 6 | **3-benzylmorphine** |  | (4R,4aR,7S,7aR,12bS)-9-benzyl-3-methyl-1,2,4,4a,7,7a,10,13-octahydro-4,12-methanobenzofuro[3,2-e]isoquinoline-7,9-diol | Y | N | N | N | Codeine-dionine family (benzylmorphine) | apparently not prescribed currently/in the past; benzylmorphine/Peronine derivative |
| 7 | **3-hydroxy-N-methylmorphinan** | Dromoran; (+-)-3-Hydroxy-N-methylmorphinan = Racemorphan; (-)-3-Hydroxy-N-methylmorphinan = Levorphanol, levo-Dromoran (see prescription drugs) | (1R,9R,10R)-17-methyl-17-azatetracyclo[7.5.3.01,10.02,7]heptadeca-2(7),3,5-trien-4-ol;hydrobromide | Y | N | N | N | Morphinan, morphinan series | see morphinan, levorphanol (L-3-Hydroxy-N-methylmorphinan), dextrorphan (d-3-Hydroxy-N-methylmorphinan) |
| 8 | **3-monoacetylmorphine** | 3-MAM; 3-acetylmorphine | [(4R,7S,12bS)-7-hydroxy-3-methyl-2,4,4a,7,7a,13-hexahydro-1H-4,12-methanobenzofuro[3,2-e]isoquinolin-9-yl] acetate | Y | N | N | N | Morphine derivative, active opiate metabolites | apparently not prescribed currently/in the past; diacetylmorphine metabolite |
| 9 | **4-cyano-2-dimethylamino-4,4-diphenylbutane** | methadone intermediate; premethadone | 4-(dimethylamino)-2,2-diphenylpentanenitrile | Y | N | N | N | Open chain | apparently not prescribed actally/in the past; methadone precursor |
| 10 | **4-phenylpiperidine-4-carboxylic acid** | Norpethidinic acid; Normeperidinic acid | 4-phenylpiperidine-4-carboxylic acid | N | N | N | N (Misuse of DrugsAct 1971 and the Misuse of Drugs Regulations 2001, UK) | Pethidine derivative | apparently not prescribed currently/in the past; pethidine metabolite/intermediate |
| 11 | **6-acetylmorphine hydrochloride** |  | 1-[(4R,4aR,7S,7aR,12bS)-7,9-dihydroxy-3-methyl-1,2,4,4a,7a,13-hexahydro-4,12-methanobenzofuro[3,2-e]isoquinolin-7-yl]ethanone;hydrochloride | N | N | N | N (Misuse of DrugsAct 1971 and the Misuse of Drugs Regulations 2001, UK) | Morphine derivative, active opiate metabolites (6-monoacetylmorphine) | see 6-monoacetylmorphine |
| 12 | **6-methylenedihydrodesoxymorphine** | 6-MDDM; 6-Methylenedihydroepoxymorphine | (4R,4aR,7aS,12bS)-3-methyl-7-methylidene-1,2,4,4a,5,6,7a,13-octahydro-4,12-methanobenzofuro[3,2-e]isoquinolin-9-ol | N | N | N | **Y** | Morphine family, desomorphine analogue | apparently not prescribed currently/in the past; desomorphine structrurally related; not really new (1960s) |
| 13 | **6-monoacetylmorphine** | 6-MAM; 6-acetylmorphine; 6-AM | [(4R,4aR,7S,7aR,12bS)-9-hydroxy-3-methyl-2,4,4a,7,7a,13-hexahydro-1H-4,12-methanobenzofuro[3,2-e]isoquinolin-7-yl] acetate | Y | N | N | N | Morphine derivative, active opiate metabolites | apparently not prescribed currently/in the past; diacetylmorphine metabolite |
| 14 | **6-nicotinoyldihydrocodeine** | Nicodicodine; 6-Nicotinoyl dihydrocodeine | (9-methoxy-3-methyl-2,4,4a,5,6,7,7a,13-octahydro-1H-4,12-methanobenzofuro[3,2-e]isoquinolin-7-yl) pyridine-3-carboxylate | Y | N | N | N | Dihydrocodeine series | apparently not prescribed currently/it can be used (not common); investigational |
| 15 | **Acetorphine** |  | [(14S,19R)-19-[(2R)-2-hydroxypentan-2-yl]-15-methoxy-5-methyl-13-oxa-5-azahexacyclo[13.2.2.12,8.01,6.02,14.012,20]icosa-8(20),9,11,16-tetraen-11-yl] acetate | Y | N | N | N | Oripavine derivative | apparently not prescribed currently/in the past; etorphine derivative; investigational |
| 16 | **Acetoxyketobemidone** | O-Acetylketobemidone | 3-(1-Methyl-4-propionyl-4-piperidinyl)phenyl acetate | N | N | N | **Y** | 4-phenylpiperidines, ketobemidones | apparently not prescribed currently/in the past; ketobemidone related; not really new (1950s) |
| 17 | **Acetylmethadol** | methadyl acetate | [6-(dimethylamino)-4,4-diphenylheptan-3-yl] acetate | Y | N | N | N | Open chain | racemic mixture: see alphacetylmethadol (α-acetylmethadol) and betacetylmethadol (β-acetylmethadol), which are racemic mixtures of levacetylmethadol (LAAM; L-α-acetylmethadol) and D-α-acetylmethadol and L-β-acetylmethadol and D-β-acetylmethadol, respectively; possibly marketed outside US |
| 18 | **AH-7921** | doxylam | 3,4-dichloro-N-[[1-(dimethylamino)cyclohexyl]methyl]benzamide | Y | Y | Y | N | others, benzamide family, similar to U-47700 | apparently not prescribed currently/in the past; discovered in the '70, recently sold oline as reasearch chemical |
| 19 | **Alfameprodine** | Alphameprodine; Alfameprodina | [(3S,4R)-3-ethyl-1-methyl-4-phenylpiperidin-4-yl] propanoate | Y | N | N | N | 4-phenylpiperidines, prodines | see meprodine and betameprodine (less used stereoisomer); meperidine analogue; investigational |
| 20 | **Allylprodine** | Alperidine | (1-methyl-4-phenyl-3-prop-2-enylpiperidin-4-yl) propanoate | Y | N | N | N | 4-phenylpiperidines, prodines | apparently not prescribed currently/in the past; pethidine related; investigational |
| 21 | **Alpha-3-acetoxy-6-methylanino-4,4-diphenylheptane** |  |  | N | N | N | N (Misuse of Drugs Act 1971 and the Misuse of Drugs Regulations 2001, UK) | Methadone related | apparently not prescribed currently/in the past; methadone related |
| 22 | **Alphacetylmethadol** | α-acetylmethadol; AAM; Alphacemethadone | [(3R,6R)-6-(dimethylamino)-4,4-diphenylheptan-3-yl] acetate | Y | N | N | N | Open chain | apparently not prescribed currently/never marketed in US; levacetylmethadol enantiomer (similar in structure to methadone); withdrawn |
| 23 | **Alphamethadol** |  | (3R,6R)-6-(dimethylamino)-4,4-diphenylheptan-3-ol | Y | N | N | N | Open chain, dimepheptadol isomer | apparently not prescribed currently/in the past; dimepheptadol/methadol isomer (the other being betamethadol) composed of two isomers itself (L-α-methadol, and D-α-methadol); investigational |
| 24 | **Alphaprodine** |  | (1,3-dimethyl-4-phenylpiperidin-4-yl) propanoate | Y | N | N | N | 4-phenylpiperidines, prodines | apparently not prescribed currently/US previously marketed; prodine isomer (see also betaprodine); desomorphine structurally related; first approved in 1949 |
| 25 | **BDPC** | Bis(2,4-dinitrophenyl)carbonate; bromadol | bis(2,4-dinitrophenyl) carbonate | N | N | N | **Y** | others | apparently not prescribed currently/in the past; arylcyclohexylamine chemical structure; not really new (1970s), seized in Canada (2013), scheduled in Minnesota |
| 26 | **Benzethidine** |  | ethyl 4-phenyl-1-(2-phenylmethoxyethyl)piperidine-4-carboxylate | Y | N | N | N | 4-phenylpiperidine derivative; pethidine related | apparently not prescribed currently/in the past; pethidine related; investigational |
| 27 | **Benzylmorphine myristate** | Myrophine; Myristylbenzylmorphine; | [(4R,4aR,7S,7aR,12bS)-3-methyl-9-phenylmethoxy-2,4,4a,7,7a,13-hexahydro-1H-4,12-methanobenzofuro[3,2-e]isoquinolin-7-yl] tetradecanoate | Y | N | N | N | Morphine derivative, codeine-dionine family (benzylmorphine) | apparently not prescribed currently/in the past; morphine derivative; investigational |
| 28 | **Betacetylmethadol** |  | [(3S,6R)-6-(dimethylamino)-4,4-diphenylheptan-3-yl] acetate | Y | N | N | N | Open chain, alpha/levoacetylmethadol isomer | apparently not prescribed currently/in the past; alpha/levoacetylmethadol isomer; investigational |
| 29 | **Betameprodine** |  | [(3R,4R)-3-ethyl-1-methyl-4-phenylpiperidin-4-yl] propanoate | Y | N | N | N | 4-phenylpiperidines, prodines, meprodine isomer | see meprodine and alfameprodine (more used stereoisomer); meperidine analogue |
| 30 | **Betamethadol** | β-methadol; betametadol | (3S,6R)-6-(dimethylamino)-4,4-diphenylheptan-3-ol | Y | N | N | N | Open chain, dimepheptadol isomer | may be available in some countries; dimepheptadol/methadol isomer (the other being alphamethadol) composed of two isomers itself, L-β-methadol, and D-β-methadol; investigational |
| 31 | **Betaprodine** |  | [(3R,4R)-1,3-dimethyl-4-phenylpiperidin-4-yl] propanoate | Y | N | N | N | Phenipiperidine - prodine isomer | apparently not prescribed currently/it has been used in the past; prodine isomer (as well as alphaprodine); investigational |
| 32 | **Bromadoline** | U-47931E | 4-bromo-N-[(1S,2S)-2-(dimethylamino)cyclohexyl]benzamide | N | Y | Y | N | others | apparently not prescribed currently/in the past; developed in the '70; AH-7921 and U-47700 related; investigatonal |
| 33 | **Carperidine** |  | ethyl 1-(3-amino-3-oxopropyl)-4-phenylpiperidine-4-carboxylate | N | N | N | **Y** | 4-phenylpiperidines, pethidines (meperidines) | apparently not prescribed currently/in the past; 4-phenylpiperidine derivative; investigational; not really new, already known to legislations, specifically excluded from the illegal drug schedules |
| 34 | **Clonitazene** |  | 2-[2-[(4-chlorophenyl)methyl]-5-nitrobenzimidazol-1-yl]-N,N-diethylethanamine | Y | N | N | N | Benzimidazoles | apparently not prescribed currently/in the past; etonitazene related; investigational |
| 35 | **Codeine methylbromide** | eucodeine; Eucodin; methobromide (salt) |  | N | N | N | N (US Controlled Substances Act) | Codeine derivative | apparently not prescribed currently/previously marketed ad eucodine; codeine salt; illicit |
| 36 | **Codeine-N-oxide** | genocodeine | (3S,4R,4aR,7S,7aR,12bS)-9-methoxy-3-methyl-3-oxido-2,4,4a,7,7a,13-hexahydro-1H-4,12-methanobenzofuro[3,2-e]isoquinolin-3-ium-7-ol | Y | N | N | N | Codeine derivative, nitrogen morphine derivatives | apparently not prescribed currently/in the past; codeine metabolite; illicit |
| 37 | **Codoxime** | Codossima; Dihydrocodeinone-6-carboxymethyloxime | 2-[(E)-[(4R,4aR,7aR,12bS)-9-methoxy-3-methyl-1,2,4,4a,5,6,7a,13-octahydro-4,12-methanobenzofuro[3,2-e]isoquinolin-7-ylidene]amino]oxyacetic acid | Y | N | N | N | Morphinones and morphols | apparently not prescribed currently/in the past; hydrocodone derivative; investigational |
| 38 | **Cyclazocine** |  | 10-(cyclopropylmethyl)-1,13-dimethyl-10-azatricyclo[7.3.1.02,7]trideca-2(7),3,5-trien-4-ol | N | N | N | **Y** | Opioid antagonists and inverse agonists, mixed opioid agonist/antagonist | apparently not prescribed currently/in the past; dezocine, pentazocine and phenazocine related; investigational; not really new (1960s), specifically excluded from the illegal drug schedules |
| 39 | **Cyprenorphine** | M-285 | 5-(cyclopropylmethyl)-19-(2-hydroxypropan-2-yl)-15-methoxy-13-oxa-5-azahexacyclo[13.2.2.12,8.01,6.02,14.012,20]icosa-8(20),9,11,16-tetraen-11-ol | N | N | N | N (US Controlled Substances Act) | Oripavine derivative | apparently not prescribed currently/in the past; buprenorphine related; investigational |
| 40 | **D3-Codeine** | Codeine-d3 solution | (4R,4aR,7S,7aR,12bS)-9-methoxy-3-(trideuteriomethyl)-2,4,4a,7,7a,13-hexahydro-1H-4,12-methanobenzofuro[3,2-e]isoquinolin-7-ol | N | N | N | N (Misuse of DrugsAct 1971 and the Misuse of Drugs Regulations 2001, UK) | Codeine derivative, codeine-dionine family | apparently not prescribed currently/in the past; codeine derivative; deuterated international standard |
| 41 | **D3-Morphine** | Deuteriomorphine; morphine-d3 | (4R,4aR,7S,7aR,12bS)-3-(trideuteriomethyl)-2,4,4a,7,7a,13-hexahydro-1H-4,12-methanobenzofuro[3,2-e]isoquinoline-7,9-diol | N | N | N | N (Misuse of DrugsAct 1971 and the Misuse of Drugs Regulations 2001, UK) | Morphine derivative | apparently not prescribed currently/in the past; morphine derivative; deuterated international standard |
| 42 | **Demethylmorphine** | Desmethylmorphine; Normorphine | 1,2,3,4,4a,7,7a,13-octahydro-4,12-methanobenzofuro[3,2-e]isoquinoline-7,9-diol | Y | N | N | N | Morphine derivative | apparently not prescribed currently/in the past; morphine metabilite/derivative; and intermediate; investigational |
| 43 | **Desmetramadol** | O-desmethyltramadol; ODT; O-DSMT; Omnitram; Krypton (powdered kratom leaf laced with desmetramadol) | 3-[2-[(dimethylamino)methyl]-1-hydroxycyclohexyl]phenol | N | Y | Y | N | others, tramadol metabolite | apparently not prescribed currently/in the past; tramadol metabolite; investigational |
| 44 | **Desomorphine** | krokodil; dihydrodesoxymorphine; Permonid | (4R,4aR,7aS,12bS)-3-methyl-2,4,4a,5,6,7,7a,13-octahydro-1H-4,12-methanobenzofuro[3,2-e]isoquinolin-9-ol | Y | N | N | N | Morphine family | not prescribed currently/has been prescribed in the past; morphine analogue; investigational |
| 45 | **Dextrorphan** | DXO; Levorphanol d-form | (1S,9S,10S)-17-methyl-17-azatetracyclo[7.5.3.01,10.02,7]heptadeca-2(7),3,5-trien-4-ol | Dextromethorphan and dextrorphan are not under international control. | N | N | N | Dextromethorphan metabolite | apparently not prescribed currently/in the past; dextromethorphan metabolite; investigational |
| 46 | **Diampromide** |  | N-[2-[methyl(2-phenylethyl)amino]propyl]-N-phenylpropanamide | Y | N | N | N | Open chain | apparently not prescribed currently/in the past; propiram and phenampromide related; ring-opened analogue of fentanyl; investigational |
| 47 | **Diethylthiambutene** | Thiambutene; Themalon; Diethibutin | N,N-diethyl-4,4-dithiophen-2-ylbut-3-en-2-amine | Y | N | N | N | Open chain | used in veterinary medicine; dimethylthiambutene and ethylmethylthiambutene related; investigational |
| 48 | **Dihydroetorphine** | DHE | (1S,2S,6R,14R,15R,16R)-16-[(2R)-2-hydroxypentan-2-yl]-15-methoxy-5-methyl-13-oxa-5-azahexacyclo[13.2.2.12,8.01,6.02,14.012,20]icosa-8(20),9,11-trien-11-ol | Y | N | N | N | Oripavine derivative | may be available in some countries; etorphine derivative; |
| 49 | **Dihydromorphine** | Paramorfan; Paramorphan | (4R,4aR,7S,7aR,12bS)-3-methyl-2,4,4a,5,6,7,7a,13-octahydro-1H-4,12-methanobenzofuro[3,2-e]isoquinoline-7,9-diol | Y | N | N | N | Morphine derivative | may be available in some countries; dihydrocodeine metabolite; may occurr in opium; [3H]-dihydromorphine used for research purposes |
| 50 | **Dihydromorphine 3,6-diglucuronide** |  |  | N | N | N | N (Misuse of DrugsAct 1971 and the Misuse of Drugs Regulations 2001, UK) | Dihydromorphine derivative | apparently not prescribed currently/in the past; opioid metabolite; |
| 51 | **Dimenoxadol** |  | 2-(dimethylamino)ethyl 2-ethoxy-2,2-diphenylacetate | Y | N | N | N | Open chain | may be available in some countries; methadone and dextropropoxyphene structurally related; investigational |
| 52 | **Dimepheptanol** | Amidol; Pangerin; methadol; racemethadol; imethadol; betamethadol | 6-(dimethylamino)-4,4-diphenylheptan-3-ol | Y | N | N | N | Open chain, methadone related | apparently not prescribed currently/in the past; two isomers mixture, alphamethadol (α-methadol) and betamethadol (β-methadol); each of these isomers is itself a mixture of two isomers; methadone related; investigational |
| 53 | **Dimethylthiambutene** | DMTB; Ohton; Aminobutene; Dimethibutin; Kobaton; Takaton; Dimethibutin | N,N-dimethyl-4,4-dithiophen-2-ylbut-3-en-2-amine | Y | N | N | N | Open chain | may be available in some countries; often used in veterinary medicine; diethylthiambutene and ethylmethylthiambutene related; investigational |
| 54 | **Dioxaphetyl butyrate** | Amidalgon; Spasmoxal | ethyl 4-morpholin-4-yl-2,2-diphenylbutanoate | Y | N | N | N | Open chain | apparently not prescribed currently/in the past; dextropropoxyphene, levacetylmethadol (LAAM), lefetamine and dimenoxadol; investigational |
| 55 | **Dipipanone** | Pipadone; Diconal (commercially available, dipipanone hydrochloride mixed with cyclizine hydrochloride) | 4,4-diphenyl-6-piperidin-1-ylheptan-3-one | Y | N | N | N | Open chain | may be available in some countries; methadone and phenaxodone structurally related; possibly marketed outside US |
| 56 | **Drotebanol** | Oxymethebanol; Metebanyl | (1R,9R,10S,13R)-3,4-dimethoxy-17-methyl-17-azatetracyclo[7.5.3.01,10.02,7]heptadeca-2(7),3,5-triene-10,13-diol | Y | N | N | N | Morphinan, others | may be available in some countries; thebaine derivative; possibily marketed outside US |
| 57 | **Embutramide** | Embutane; T61, Tanax (with Mebezonium Iodide and Tetracaine); Tributame (with chloroquine and lidocaine) | N-[2-ethyl-2-(3-methoxyphenyl)butyl]-4-hydroxybutanamide | N | N | N | N (US Controlled Substances Act) | Open chain | used in veterinary medicine; methadone structurally related |
| 58 | **Ethoheptazine** | Zactane; Equagesic (commercially available, ethoheptazine citrate mixed with acetylsalicylic acid and meprobamate) | ethyl 1-methyl-4-phenylazepane-4-carboxylate | Y | N | N | N | Phenazepanes | available in some countries (as combination with acetylsalicylic acid and meprobamate); proheptazine and pethidine related; withdrawn in US |
| 59 | **Ethylmethylthiambutene** | Emethibutin | N-ethyl-N-methyl-4,4-dithiophen-2-ylbut-3-en-2-amine | Y | N | N | N | Open chain | apparently not prescribed currently/in the past; thiambutene family; investigational |
| 60 | **Etonitazene** |  | 2-[2-[(4-ethoxyphenyl)methyl]-5-nitrobenzimidazol-1-yl]-N,N-diethylethanamine | Y | N | N | N | Benzimidazoles | apparently not prescribed currently/in the past; clonitazene structurally related; investigational |
| 61 | **Etorphine** | M99; Immobilon | (1R,2S,6R,14R,15R,19R)-19-[(2R)-2-hydroxypentan-2-yl]-15-methoxy-5-methyl-13-oxa-5-azahexacyclo[13.2.2.12,8.01,6.02,14.012,20]icosa-8(20),9,11,16-tetraen-11-ol | Y | N | N | N | Oripavine derivative | used in veterinary medicine; oripavine derivative |
| 62 | **Etorphine hydrochloride** |  | (5α,7α)-7-[(2R)-2-Hydroxy-2-pentanyl]-6-methoxy-17-methyl-4,5-epoxy-6,14-ethenomorphinan-3-ol hydrochloride (1:1) | Y | N | N | N | Oripavine derivative | used in veterinary medicine; oripavine derivative |
| 63 | **Etoxeridine** | Carbetidine; Atenos | ethyl 1-[2-(2-hydroxyethoxy)ethyl]-4-phenylpiperidine-4-carboxylate | Y | N | N | N | 4-phenylpiperidine derivative, pethidine related | apparently not prescribed currently/in the past; pethidine related; investigational |
| 64 | **Furethidine** |  | ethyl 1-[2-(oxolan-2-ylmethoxy)ethyl]-4-phenylpiperidine-4-carboxylate | Y | N | N | N | 4-phenylpiperidine derivative, pethidine related | apparently not prescribed currently/in the past; pethidine related; investigational |
| 65 | **Hexadeuterated diamorphine** |  |  | N | N | N | N (Misuse of DrugsAct 1971 and the Misuse of Drugs Regulations 2001, UK) | international standard ligand | apparently not prescribed currently/in the past; international standard ligand; investigational |
| 66 | **Hydromorphinol** | RAM-320; 14-hydroxydihydromorphine; α-oxymorphol | (4R,4aS,7S,7aR,12bS)-3-methyl-1,2,4,5,6,7,7a,13-octahydro-4,12-methanobenzofuro[3,2-e]isoquinoline-4a,7,9-triol | Y | N | N | N | Morphine family | apparently not prescribed currently/in the past; morphine derivative; investigational |
| 67 | **Hydroxypethidine** | Bemidone | ethyl 4-(3-hydroxyphenyl)-1-methylpiperidine-4-carboxylate | Y | N | N | N | 4-phenylpiperidines, pethidines (meperidines) | apparently not prescribed currently/in the past; pethidine related; investigational |
| 68 | **Isocodeine** | 6-Isocodeine; α-Isocodeine | (4R,4aR,7R,7aR,12bS)-9-methoxy-3-methyl-2,4,4a,7,7a,13-hexahydro-1H-4,12-methanobenzofuro[3,2-e]isoquinolin-7-ol | N | N | N | N (Misuse of DrugsAct 1971 and the Misuse of Drugs Regulations 2001, UK) | Codeine-dionine family | apparently not prescribed currently/in the past; codeine derivative; oppioid research chemical |
| 69 | **Isomethadone** | Liden; isoamidone | 6-(dimethylamino)-5-methyl-4,4-diphenylhexan-3-one | Y | N | N | N | Open chain | apparently not prescribed currently/has been peviously marketed; metahdone related; investigational |
| 70 | **Isopropyl-U-47700** | Isopropyl-U47; IP-U47 | trans-3,4-dichloro-N-2-(dimethylamino)cyclohexyl)-N-isopropylbenzamide | N | Y | Y | N | others, U-47700 derivative | not prescribed currently/in the past; U-47700 related; recently sold as research chemical |
| 71 | **Lefetamine** | SPA; Santenol | (1R)-N,N-dimethyl-1,2-diphenylethanamine | Y | N | N | N | Open chain | may be available in some countries; open chain; possibly marketed outside US |
| 72 | **Levallorphan** | levallorphan tartrate; Lorfan; Naloxifan; Naloxiphan; Pethilorfan ( combination of levallorphan with pethidine (meperidine)) | (1R,9R,10R)-17-prop-2-enyl-17-azatetracyclo[7.5.3.01,10.02,7]heptadeca-2(7),3,5-trien-4-ol | N | N | N | N (approved prescribed drug) | Opioid antagonists and inverse agonists, opioid modulator, morphinan family | available in some countries; morphinan family; US previously marketed |
| 73 | **Levargorphan** |  | l-11-propargyl-1,2,3,9,10,10a-hexahydro-4H-10,4a-iminoethanophenanthren-6-ol | N | N | N | N (Controlled Drugs and Substances Act, Canada) | Morphinan, morphinan series | apparently not prescribed currently/in the past; morphinan |
| 74 | **Levomethorphan** |  | (1R,9R,10R)-4-methoxy-17-methyl-17-azatetracyclo[7.5.3.01,10.02,7]heptadeca-2(7),3,5-triene | Y | N | N | N | Morphinan, morphinan series | apparently not prescribed currently/in the past; morphinan; levorphanol prodrug; investigational |
| 75 | **Levomoramide** |  | (3R)-3-methyl-4-morpholin-4-yl-2,2-diphenyl-1-pyrrolidin-1-ylbutan-1-one | Y | N | N | N | Open chain | apparently not prescribed currently/in the past; dextromoramide inactive isomer; possibly marketed outside US |
| 76 | **Levophenacylmorphan** | Benzorphanol | 2-[(1R,9R,10R)-4-hydroxy-17-azatetracyclo[7.5.3.01,10.02,7]heptadeca-2(7),3,5-trien-17-yl]-1-phenylethanone | Y | N | N | N | Morphinan, morphinan series | apparently not prescribed currently/in the past; morphinanan; investigational |
| 77 | **Levorphanol** | L-Dromoran; Levo-Dromoran; Ro 1-5431 | (1R,9R,10R)-17-methyl-17-azatetracyclo[7.5.3.01,10.02,7]heptadeca-2(7),3,5-trien-4-ol | Y | N | N | N | Morphinans, morphinan series. One of four enantiomers of the compound racemorphan. | available in some countries; see racemorphan; US approved Rx |
| 78 | **Meprodine** | (α/β)-Meprodine | see Alphameprodine, Betameprodine | Y | N | N | N | 4-Phenylpiperidines, prodines, pethidine analogue, prodine related | see alphameprodine, betameprodine; illicit |
| 79 | **Metazocine** |  | 1,10,13-trimethyl-10-azatricyclo[7.3.1.02,7]trideca-2(7),3,5-trien-4-ol | Y | N | N | N | Benzomorphan derivatives, pentazocine related, | apparently not prescribed currently/in the past; pentazocine related; investigational |
| 80 | **Metethoheptazine** | WY-535 | ethyl 1,3-dimethyl-4-phenylazepane-4-carboxylate | N | N | N | **Y** | phenazepanes | apparently not prescribed currently/in the past; phenazepine family; investigational; not really new (1960s), specifically excluded from the illegal drug schedules |
| 81 | **Metheptazine** |  | methyl 1,2-dimethyl-4-phenylazepane-4-carboxylate | N | N | N | **Y** | Phenazepines | apparently not prescribed currently/in the past; phenazepine family; investigational; not really new (1950s), specifically excluded from the illegal drug schedules |
| 82 | **Methorphan** | dextromethorphan; levomethorphan; racemethorphan | 4-methoxy-17-methyl-17-azatetracyclo[7.5.3.01,10.02,7]heptadeca-2(7),3,5-triene | Y | N | N | N | Morphinan family, two isomeric forms, each with differing pharmacology and effects | two isomeric forms: see dextromethorphan (OTC cough suppressant) and levomethorphan; see racemethorphan |
| 83 | **Methyldihydromorphine** | dihydroheterocodeine | (4R,4aR,7S,7aR,12bS)-3,7-dimethyl-1,2,4,4a,5,6,7a,13-octahydro-4,12-methanobenzofuro[3,2-e]isoquinoline-7,9-diol | Y | N | N | N | Codeine-dionine family, heterocodeine derivative | apparently not prescribed currently/in the past; hydromorphone derivative; investtigational |
| 84 | **Metopon** | Methyldihydromorphinone | (4R,4aR,7aR,12bS)-9-hydroxy-3,7a-dimethyl-2,4,4a,5,6,13-hexahydro-1H-4,12-methanobenzofuro[3,2-e]isoquinolin-7-one | Y | N | N | N | Morphinones and morphols | may be available in some countries; hydromorphone derivative; US peviously marketed |
| 85 | **Moramide intermediate** | 2-Methyl-3-morpholino-1,1-diphenylpropane-carboxylic acid | 3-methyl-4-morpholin-4-yl-2,2-diphenylbutanoic acid | Y | N | N | N | Open chain | apparently not prescribed currently/in the past; moramide precursor |
| 86 | **Morphanol** | Racemorphan (Dextrorphan and Levorphan); Morphinan-3-ol, 17-methyl-, (+/-)- | 17-methyl-17-azatetracyclo[7.5.3.01,10.02,7]heptadeca-2(7),3,5-trien-4-ol | Y | N | N | N | Morphinan, morphinan series | racemic mixture: see dextrorphan and levorphanol; US previously marketed |
| 87 | **Morpheridine** | Morpholinoethylnorpethidine | ethyl 1-(2-morpholin-4-ylethyl)-4-phenylpiperidine-4-carboxylate | Y | N | N | N | 4-phenylpiperidine derivative, pethidine related | apparently not prescribed currently/in the past; pethidine related; investigational |
| 88 | **Morphine Methobromide** | Morphine methylbromide; Morphine bromomethylate; Morphosan | (4R,4aR,7S,7aR,12bS)-3,3-dimethyl-2,4,4a,7,7a,13-hexahydro-1H-4,12-methanobenzofuro[3,2-e]isoquinolin-3-ium-7,9-diol;bromide | Y | N | N | N | Morphine derivative | see morphine; morphine derivative (salt) |
| 89 | **Morphine methylsulfonate** |  | [(4R,4aR,7S,7aR,12bS)-7-hydroxy-3-methyl-2,4,4a,7,7a,13-hexahydro-1H-4,12-methanobenzofuro[3,2-e]isoquinolin-9-yl] methanesulfonate | Y | N | N | N | Morphine derivative | see morphine; morphine derivative (salt) |
| 90 | **Morphine N-oxide** | Morphine-N-Oxide; genomorphine | (4R,4aR,7S,7aR,12bS)-3-methyl-3-oxido-2,4,4a,7,7a,13-hexahydro-1H-4,12-methanobenzofuro[3,2-e]isoquinolin-3-ium-7,9-diol | Y | N | N | N | Nitrogen morphine derivatives | apparently not prescribed currently/in the past; morphine metabolite; morphine decomposition product (also in poppy) |
| 91 | **Morphine3, 6-diacalte** |  |  | N | N | N | N (Misuse of DrugsAct 1971 and the Misuse of Drugs Regulations 2001, UK) | Morphine derivative | apparently not prescribed currently/in the past; morphine derivative |
| 92 | **Morphine-3-B-D-Glucoride-N-oxide** | Morphine 3-β-D-glucuronide-N-Oxide |  | N | N | N | N (Misuse of DrugsAct 1971 and the Misuse of Drugs Regulations 2001, UK) | Morphine derivative | apparently not prescribed currently/in the past; morphine derivative/metabolite |
| 93 | **Morphine-3-B-D-Glucuronide** | Morphine-3-glucuronide; Morphine-3-beta-D-glucuronide | (2S,3S,4S,5R,6S)-6-[[(4R,4aR,7S,7aR,12bS)-7-hydroxy-3-methyl-2,4,4a,7,7a,13-hexahydro-1H-4,12-methanobenzofuro[3,2-e]isoquinolin-9-yl]oxy]-3,4,5-trihydroxyoxane-2-carboxylic acid | N | N | N | N (Misuse of DrugsAct 1971 and the Misuse of Drugs Regulations 2001, UK) | Morphine derivative | apparently not prescribed currently/in the past; morphine derivative/metabolite |
| 94 | **Morphine-6-B-D-glucuronide-N-oxide** |  |  | N | N | N | N (Misuse of DrugsAct 1971 and the Misuse of Drugs Regulations 2001, UK) | Morphine derivative | apparently not prescribed currently/in the past; morphine derivative/metabolite |
| 95 | **Morphine-6-sulphate** | M6S |  | N | N | N | N (Misuse of DrugsAct 1971 and the Misuse of Drugs Regulations 2001, UK) | Morphine derivative | apparently not prescribed currently/in the past; morphine derivative/metabolite |
| 96 | **MT-45** | IC-6 | 1-Cyclohexyl-4-(1,2-diphenylethyl)piperazine | Y | Y | N | N | others, piperazine family | not prescribed currently/in the past; piperazine derivative; recently sold as research chemical |
| 97 | **N-benzylpethidine** |  |  | N | N | N | N (Misuse of DrugsAct 1971 and the Misuse of Drugs Regulations 2001, UK) | Pethidine derivative | apparently not prescribed currently/in the past; pethidine derivative |
| 98 | **N-Demethylcodeine** | Norcodeine | (4R,4aR,7S,7aR,12bS)-9-methoxy-1,2,3,4,4a,7,7a,13-octahydro-4,12-methanobenzofuro[3,2-e]isoquinolin-7-ol | Y | N | N | N | Active opiate metabolites | apparently not prescribed currently/in the past; codeine metabolite; investigational |
| 99 | **Nicocodeine** | Nicocodine; Lyopect; 6-Nicotinoylcodeine | [(4R,4aR,7S,7aR,12bS)-9-methoxy-3-methyl-2,4,4a,7,7a,13-hexahydro-1H-4,12-methanobenzofuro[3,2-e]isoquinolin-7-yl] pyridine-3-carboxylate | Y | N | N | N | Dihydrocodeine series | may be available in some countries (salt); codeine derivative; dihydrocodeine and nicomorphine related; investigational |
| 100 | **Nicodicodeine** | 6-Nicotinoyl dihydrocodeine | [(4R,4aR,7S,7aR,12bS)-9-methoxy-3-methyl-2,4,4a,5,6,7,7a,13-octahydro-1H-4,12-methanobenzofuro[3,2-e]isoquinolin-7-yl] pyridine-3-carboxylate | Y | N | N | N | Dihydrocodeine series | apparently not prescribed currently/in the past; dihydrocodeine series |
| 101 | **Noracymethadol** | Paracymethadol; | [6-(methylamino)-4,4-diphenylheptan-3-yl] acetate | Y | N | N | N | Open chain, methadone related | apparently not prescribed currently/in the past; methadone related; investigational |
| 102 | **Norlevorphanol** | 3-Hydroxymorphinan | (1R,9R,10R)-17-azatetracyclo[7.5.3.01,10.02,7]heptadeca-2(7),3,5-trien-4-ol | Y | N | N | N | Morphinan, morphinan series | apparently not prescribed currently/in the past; hydroxymorphinan/morphinan-3-ol isomer; investigational |
| 103 | **Normorphine hemisuccinide** |  |  | Y (normorphine) | N | N | N (Misuse of DrugsAct 1971 and the Misuse of Drugs Regulations 2001, UK) | Normorphine derivative | apparently not prescribed currently/in the past; normorphine is a morphine derivative, a morphine metabolite and a opioid synthesis intermediate; |
| 104 | **Norpethidine** | normeperidine; pethidine intermediate B | ethyl 4-phenylpiperidine-4-carboxylate | Y | N | N | N | 4-phenylpiperidines, pethidines (meperidines) | see pethidine; pethidine metabolite |
| 105 | **Norpipanone** | Hexalgon | 4,4-diphenyl-6-piperidin-1-ylhexan-3-one | Y | N | N | N | Open chain | may be available in some countries (as hydrochloride and hydrobromide salt); methadone related |
| 106 | **NPP** | 1-Phenethyl-4-piperidone; N-Phenethyl-4-piperidinone | 1-(2-phenylethyl)piperidin-4-one | Y | N | N | N | 4-piperidinone derivative | apparently not prescribed currently/in the past; fentanyl precursor; |
| 107 | **Oripavine** |  | (4R,7aR,12bS)-7-methoxy-3-methyl-2,4,7a,13-tetrahydro-1H-4,12-methanobenzofuro[3,2-e]isoquinolin-9-ol | Y | N | N | N | Opium and poppy straw derivatives, opium alkaloids | apparently not prescribed currently/in the past; opium alkaloid; opioid synthesis precursor |
| 108 | **Oxpheneridine** | Carbamethidine | ethyl 1-(2-hydroxy-2-phenylethyl)-4-phenylpiperidine-4-carboxylate | N | N | N | **Y** | 4-phenylpiperidine derivative, pethidine related | apparently not prescribed currently/in the past; pethidine related; investigational; not really new, already known to legislations, specifically excluded from the illegal drug schedules |
| 109 | **Oxymorphone** | Numorphan (oxymorphone hydrochloride); 14-Hydroxydihydromorphinone | (4R,4aS,7aR,12bS)-4a,9-dihydroxy-3-methyl-2,4,5,6,7a,13-hexahydro-1H-4,12-methanobenzofuro[3,2-e]isoquinolin-7-one | Y | N | N | N | Morphinones and morphols | available as prescription drug; thebaine derivative |
| 110 | **oxymorphone-3-glucuronide** |  | (2S,3S,4S,5R,6S)-6-[[(4R,4aS,7aR,12bS)-4a-hydroxy-3-methyl-7-oxo-2,4,5,6,7a,13-hexahydro-1H-4,12-methanobenzofuro[3,2-e]isoquinolin-9-yl]oxy]-3,4,5-trihydroxyoxane-2-carboxylic acid | N | N | N | N (Misuse of DrugsAct 1971 and the Misuse of Drugs Regulations 2001, UK) | Oxymorhone derivative | apparently not prescribed currently/in the past; oxymorphone metabolite; |
| 111 | **PEPAP** | synthetic heroin; 1-(2-Phenylethyl)-4-Phenyl-4-Acetoxypiperidine | 4-phenyl-1-(2-phenylethyl)piperidin-4-yl acetate | Y | N | N | N | 4-phenylpiperidines, prodines, MPPP related | apparently not prescribed currently/in the past; pethidine analog, MPPP related; |
| 112 | **Pethidine intermediate A** |  | 1-Methyl-4-phenylpiperidine-4-carbonitrile | Y | N | N | N | 4-phenylpiperidine derivative, pethidine precursor | apparently not prescribed currently/in the past; pethidine precursor |
| 113 | **Pethidinic Acid** | meperidinic acid; pethidine intermediate C | 1-methyl-4-phenylpiperidine-4-carboxylic acid | Y | N | N | N | 4-phenylpiperidine derivative, pethidine precursor and metabolite | apparently not prescribed currently/in the past;pethidine precursor and metabolite |
| 114 | **Phenadoxone** | Heptalgin; Morphidone; Heptazone | 6-morpholin-4-yl-4,4-diphenylheptan-3-one | Y | N | N | N | Open chain | may be available in some countries; open chain; investigational |
| 115 | **Phenampromide** |  | N-phenyl-N-(1-piperidin-1-ylpropan-2-yl)propanamide | Y | N | N | N | Open chain | apparently not prescribed currently/in the past; propiram and diampromide related, investigational |
| 116 | **Phenomorphan** |  | (1R,9R,10R)-17-(2-phenylethyl)-17-azatetracyclo[7.5.3.01,10.02,7]heptadeca-2(7),3,5-trien-4-ol | Y | N | N | N | Morphinan, morphinan series | apparently not prescribed currently/in the past; morphinan; investigational |
| 117 | **Piminodine** | Alvodine | ethyl 1-(3-anilinopropyl)-4-phenylpiperidine-4-carboxylate | Y | N | N | N | 4-phenylpiperidines, pethidines (meperidines) | apparently not prescribed currently/has been used in the past; pethidine analog; US previoulsy marketed |
| 118 | **Piperidylthiambutene** | Piperidino-ohton; Piperidinohton; 3-Piperidino-1,1-di(2-thienyl)but-1-ene | 1-(4,4-dithiophen-2-ylbut-3-en-2-yl)piperidine | N | N | N | **Y** | Open chain | apparently not prescribed currently/in the past; thiambutene family; not really new (1950s), sold as a designer drug (first appearing in late 2018) |
| 119 | **Proheptazine** |  | (1,3-dimethyl-4-phenylazepan-4-yl) propanoate | Y | N | N | N | Phenazepanes | apparently not prescribed currently/in the past; phenazepine family; investigational |
| 120 | **Properidine** | Ipropethidine | propan-2-yl 1-methyl-4-phenylpiperidine-4-carboxylate | Y | N | N | N | 4-phenylpiperidines, pethidine analogue | may be available in some countries (as hydrochloride salt); pethidine related; investigational |
| 121 | **Propiram** | Algeril; Dirame; Bay 4503 | N-(1-piperidin-1-ylpropan-2-yl)-N-pyridin-2-ylpropanamide | Y | N | N | N | Open chain | may be available in some countries (as fumarate salt); phenampromide and diampromide; possibly marketed outside US |
| 122 | **Racemethorphan** | see methorphan | (1R,9R)-4-methoxy-17-methyl-17-azatetracyclo[7.5.3.01,10.02,7]heptadeca-2(7),3,5-triene | Y | N | N | N | Morphinans, morphinan series | racemic mixture: see dextromethorphan (OTC cough suppressant) and levomethorphan; see methorpan |
| 123 | **Racemoramide** | moramide | racemic mixture | Y | N | N | N | Open chain | racemic mixture: dextromoramide (active) and levomoramide (inactive); investigational |
| 124 | **Salvinorin A** |  | methyl (2S,4aR,6aR,7R,9S,10aS,10bR)-9-acetyloxy-2-(furan-3-yl)-6a,10b-dimethyl-4,10-dioxo-2,4a,5,6,7,8,9,10a-octahydro-1H-benzo[f]isochromene-7-carboxylate | N | N | N | **Y** | Plant derivative, terpenoid | apparently not prescribed currently/in the past; salvia divinorum terpenoid/possible synthesis; investigational; not really new, prohibited in Australia, scheduled in Canada |
| 125 | **Salvinorin B ethoxymethyl ether** | Symmetry | methyl (2S,4aR,6aR,7R,9S,10aS,10bR)-9-(ethoxymethoxy)-2-(furan-3-yl)-6a,10b-dimethyl-4,10-dioxo-2,4a,5,6,7,8,9,10a-octahydro-1H-benzo[f]isochromene-7-carboxylate | N | N | N | **Y** | Plant derivative, others | apparently not prescribed currently/in the past; salvinorin derivative; investigational |
| 126 | **Salvinorin B methoxymethyl ether** |  | methyl (2S,4aR,6aR,7R,9S,10aS,10bR)-2-(furan-3-yl)-9-(methoxymethoxy)-6a,10b-dimethyl-4,10-dioxo-2,4a,5,6,7,8,9,10a-octahydro-1H-benzo[f]isochromene-7-carboxylate | N | N | N | **Y** | Plant derivative, others | apparently not prescribed currently/in the past; salvinorin derivative; investigational; not really new (2000s) research drug |
| 127 | **Thebaine** |  | (4R,7aR,12bS)-7,9-dimethoxy-3-methyl-2,4,7a,13-tetrahydro-1H-4,12-methanobenzofuro[3,2-e]isoquinoline | Y | N | N | N | Opium and poppy straw derivatives, opium alkaloids | apparently not prescribed currently/in the past; opium alkaloid; used as precursor |
| 128 | **Tifluadom** |  | N-[[5-(2-fluorophenyl)-1-methyl-2,3-dihydro-1,4-benzodiazepin-2-yl]methyl]thiophene-3-carboxamide | N | N | N | **Y** | others, benzodiazepine derivative | apparently not prescribed currently/in the past; investigational; not really new (1980s) |
| 129 | **Trimeperidine** | Isopromedol | [(2S,5R)-1,2,5-trimethyl-4-phenylpiperidin-4-yl] propanoate | Y | N | N | N | 4-Phenylpiperidines, prodine | may be available in some countries; pethidine related |
| 130 | **U-47700** | Pinky; Fake morphine; U4 | 3,4-dichloro-N-[(1R,2R)-2-(dimethylamino)cyclohexyl]-N-methylbenzamide | Y | Y | Y | N | others, benzamide family, AH-7921 isomer | not prescribed currently/in the past; AH-7921 isomer; recently sold as research chemical |
| 131 | **U-48800** |  | 3,4-Dichloro-N-[(1R,2R)-2-(dimethylamino)cyclohexyl]-N-methylbenzamide | N | Y | Y | N | others, benzamide family | not prescribed currently/in the past; U family; recently sold as research chemical |
| 132 | **U-49900** |  | 3,4-dichloro-N-(2-(diethylamino)cyclohexyl)-N-methylbenzamide | N | Y | Y | N | others, benzamide family, U-47700 related | not prescribed currently/in the past; U-47700 related; recently sold as research chemical |
| 133 | **U-50488** | U-50488H; trans-3,4-Dichloro-N-methyl-N-[2-(1-pyrrolidinyl)cyclohexyl]-benzeneacetamide | 2-(3,4-dichlorophenyl)-N-methyl-N-[(1R,2R)-2-pyrrolidin-1-ylcyclohexyl]acetamide | N | Y | Y | N | others, acetamide family, U-47700 related | not prescribed currently/in the past; U-47700 related; recently sold as research chemical |
| 134 | **U-51754** | methene-U-47700 | trans-3,4-dichloro-N-[2-(dimethylamino)cyclo hexyl]-N-methyl-benzeneacetamide | N | Y | Y | N | others, acetamide family, U-47700 related | not prescribed currently/in the past; U-47700 related; recently sold as research chemical |
| 135 | **W-15** | 1-Phenylethylpiperidylidene-2-(4-chlorophenyl)sulfonamide | 4-chloro-N-[1-(2-phenylethyl)-2-piperidinylidene]-benzenesulfonamide; (NE)-4-chloro-N-[1-(2-phenylethyl)piperidin-2-ylidene]benzenesulfonamide | N | N | Y | N | others; W series | not prescribed currently/in the past; W series; recently sold as research chemical |
| 136 | **W-18** |  | 4-chloro-N-[1-[2-(4-nitrophenyl)ethyl]-2-piperidinylidene]-benzenesulfonamide; (NZ)-4-chloro-N-[1-[2-(4-nitrophenyl)ethyl]piperidin-2-ylidene]benzenesulfonamide (su pubchem) | N | Y | N | N | others; W series | not prescribed currently/in the past; W series; recently sold as research chemical |

*Table S2C – NPS.Finder® non fentanyl analogues (miscellaneous opioids) and comparison between the different databases.*
